# Trends in the hypertension care cascade under dual guideline criteria in Peru, 2014–2024: a decade of nationally representative surveys with joinpoint analysis and projections toward the WHO 2030 targets

**DOI:** 10.3389/fpubh.2026.1830773

**Published:** 2026-06-03

**Authors:** Jhosmer Ballena-Caicedo, Víctor Juan Vera-Ponce

**Affiliations:** Facultad de Medicina (FAMED), Universidad Nacional Toribio Rodríguez de Mendoza de Amazonas, Chachapoyas, Peru

**Keywords:** awareness, care cascade, control, ENDES, health inequalities, hypertension, Peru, treatment

## Abstract

**Background:**

Prior assessments of the hypertension care cascade in Peru have covered short periods and used a single diagnostic threshold.

**Objective:**

To describe 11-year trends (2014–2024) in the hypertension care cascade under two contemporary criteria (2023 ESH and 2025 ACC/AHA), quantify socioeconomic inequalities, and project the cascade to 2030 relative to WHO HEARTS targets.

**Methods:**

Repeated cross-sectional analysis of 238,738 adults (≥18 years) from the Peruvian Demographic and Family Health Survey (ENDES), 2014–2024. Hypertension was defined by ESH 2023 (≥140/90 mmHg or lifetime prior diagnosis) and ACC/AHA 2025 (≥130/80 mmHg or lifetime prior diagnosis). The cascade comprised awareness, pharmacological treatment, and control (<140/90 and <130/80 mmHg). Prevalences were age-standardized to the WHO World Standard Population. Joinpoint regression was applied to 23 time series and wealth-related inequalities were assessed with the Erreygers concentration index.

**Results:**

Age-standardized ESH prevalence did not change significantly (AAPC 0.88%; *p* = 0.261), whereas ACC/AHA prevalence increased (AAPC 1.92%; *p* = 0.022), as did guideline discordance (AAPC 2.88%; *p* = 0.002); no joinpoints were identified. In 2024 (ESH), 53.6% of hypertensive adults were aware of their diagnosis, 60.3% of those diagnosed received treatment, and 59.0% of treated individuals achieved control <140/90 mmHg; population-level treatment and control were 32.3 and 19.1%. The Erreygers index indicated stronger pro-rich inequality for treatment than for awareness. Projected 2030 values for awareness, treatment, and control were 54.2, 33.5, and 21.0%, leaving gaps of 25.8, 30.5, and 30.2 percentage points relative to 80–80-80 targets.

**Conclusion:**

Peru’s population-level hypertension care cascade showed persistent stagnation during 2014–2024. Current trends indicate the country will not achieve HEARTS 2030 targets without large-scale primary care interventions with an equity focus.

## 1 Introduction

Hypertension (HTN) is the leading modifiable risk factor for cardiovascular disease and premature death. In 2019, elevated systolic blood pressure accounted for ~19% of all deaths worldwide and ranked among the leading metabolic risks for mortality and disability (1). Although detection and treatment are feasible in primary care, the NCD Risk Factor Collaboration (NCD-RisC) estimated that the number of people aged 30–79 years with hypertension doubled between 1990 and 2019 (from ~648 million to ~1.28 billion), with 82% living in low- and middle-income countries (2). Similarly, the World Health Organization (WHO) reports that nearly four in five people with hypertension do not receive adequate treatment and that elevated systolic blood pressure is associated with more than 10 million deaths each year (3).

To identify where losses accumulate along the continuum of care, the hypertension care cascade disaggregates progression from need for care (people with hypertension) to prior diagnosis (awareness), pharmacological treatment, and blood pressure control. This framework is widely used to compare health system performance and inequities across countries and population groups, and to guide targeted interventions (4).

Socioeconomic inequalities are particularly relevant in cascade analyses because aggregate national coverage can conceal unequal access to diagnosis, medication, follow-up, and effective control. In low- and middle-income countries, hypertension prevalence, awareness, treatment, and control may follow different socioeconomic gradients, with treatment and continuity of care often more concentrated among advantaged groups. Therefore, quantifying wealth-related inequality is necessary to distinguish population-wide stagnation from inequitable distribution of effective coverage (2).

In 2016, the WHO and the U.S. Centers for Disease Control and Prevention (CDC) launched the Global Hearts initiative; within its clinical management component, the HEARTS technical package proposes evidence-based interventions to strengthen detection, protocolized treatment, access to essential medicines, and monitoring in primary care (5). A programmatic target proposed to accelerate progress is the 80–80–80 goal: 80% of people with hypertension know their diagnosis, 80% of those receive treatment, and 80% of those treated reach the control target (≈51% population-level control). Global modeling suggests that achieving this goal could avert 76–130 million deaths between 2022 and 2050, with substantial benefits in low- and middle-income countries (6). However, implementation of hypertension control programs in 32 low- and middle-income countries has shown a median population-level control of around 11%, underscoring persistent gaps in coverage, continuity, and quality of care (7).

In Peru, available studies describe the hypertension care cascade over limited periods or with partial approaches. Villarreal-Zegarra et al. analyzed trends from 2015 to 2018 and reported increasing prevalence along with declines in diagnostic awareness and control - (8). Carrillo-Larco et al. evaluated mean blood pressure across cascade stages using six national surveys (2015–2020), but did not formally model temporal changes nor compare alternative diagnostic thresholds - (9). More recently, Diaz-Arocutipa described trends in awareness, treatment, and control in ENDES 2019–2023 without incorporating the full pre-pandemic period or evaluating socioeconomic inequalities or future projections - (10).

In addition, no national analysis has systematically compared cascade trends under contemporary definitions with substantially different thresholds, such as the 2023 ESH guideline and the 2025 ACC/AHA guideline (11, 12). This comparison is relevant because adopting lower thresholds can markedly change the estimated prevalence and the proportion classified as uncontrolled, which in turn shapes interpretation of progress and resource needs (13).

Therefore, this study aims to analyze national trends (2014–2024) in the hypertension care cascade among Peruvian adults, comparing diagnostic and control definitions under the 2023 ESH and 2025 ACC/AHA guidelines, quantifying socioeconomic inequalities, and projecting indicators to 2030. This analysis seeks to inform prevention and control policies, strengthen primary care (including adoption/optimization of HEARTS-like strategies), and improve monitoring of progress toward global targets.

## 2 Methods

### 2.1 Study design

We conducted a repeated cross-sectional observational study using a secondary analysis of de-identified, publicly available microdata from the Peruvian Demographic and Family Health Survey (ENDES), covering 11 consecutive annual cycles (2014–2024) (14). Each cycle was analyzed as an independent sample of the resident population; therefore, estimates reflect population-level trends rather than longitudinal follow-up of individuals. The manuscript is reported in accordance with the STROBE statement for observational studies; the checklist is provided in Supplementary material 1 (15).

### 2.2 Data source

We performed a secondary analysis of ENDES, a national survey conducted by the National Institute of Statistics and Informatics (INEI). ENDES uses a complex, stratified, two-stage probabilistic sampling design. In the first stage, primary sampling units (PSUs) are selected with probability proportional to size. In ENDES, PSUs correspond to sampling clusters: urban conglomerates in urban areas and rural enumeration areas in rural settings. In the second stage, private households within each selected PSU are selected using systematic procedures. The unit of analysis corresponds to usual residents of private households.

The design aims to produce estimates representative at the national level, by area of residence (urban/rural), by natural region, and by each of the country’s 24 departments and the Constitutional Province of Callao.

For the hypertension analysis, we used the Health Questionnaire, administered to one person aged 15 years or older selected in each household, which includes standardized blood pressure and anthropometric measurements as well as questions on prior diagnosis and treatment. We analyzed annual cycles 2014–2024 and restricted the analysis to individuals aged 18 years or older. ENDES databases are publicly available through the INEI institutional portal (www.inei.gob.pe).

The year 2014 was selected as the starting point because it corresponds to the earliest cycle in the analytic series in which the ENDES Health Questionnaire provided a comparable adult health module for one selected person aged ≥15 years per household, including blood pressure measurement and questions on hypertension diagnosis and treatment. This allowed construction of comparable annual adult hypertension indicators throughout 2014–2024.

### 2.3 Population and sample

The target population comprised adults (≥18 years) who were usual residents of private households in Peru and were surveyed in ENDES during 2014–2024. We restricted analyses to individuals ≥18 years to ensure comparability with adult hypertension definitions using fixed cutoffs, according to the 2023 European Society of Hypertension (ESH) guideline (11) and the 2025 ACC/AHA guideline (12).

The analytic subpopulation included participants selected for the ENDES Health Questionnaire who were aged ≥18 years, had at least two recorded measurements of systolic (SBP) and diastolic blood pressure (DBP), and had valid information on lifetime prior hypertension diagnosis. No *a priori* sample size calculation was performed; we analyzed all eligible records available during the study period.

Observations were classified as outside the analytic subpopulation if they had physiologically implausible blood pressure values (SBP < 70 or >270 mmHg; DBP < 30 or >150 mmHg), inconsistent measurements (SBP ≤ DBP), missing or incomplete sampling design identifiers, missing information in covariates required for stratification and equity analyses (sex, age, urban/rural area, natural region, and wealth quintile), pregnancy at the time of the survey, or missing valid anthropometric measurements needed to calculate body mass index. For survey estimation, all records with valid design identifiers and positive weights were retained in the survey design, and estimates were obtained using subpopulation/domain analysis for the analytic sample.

### 2.4 Variables

#### 2.4.1 Hypertension definition

Hypertension was defined using SBP and DBP based on the arithmetic mean of the two SBP and DBP measurements obtained during the household visit. Two operational definitions were used for comparative purposes: (i) ESH 2023 criterion: hypertension if SBP ≥ 140 mmHg and/or DBP ≥ 90 mmHg, or self-reported lifetime prior diagnosis of arterial hypertension/high blood pressure by a health professional (11); and (ii) ACC/AHA 2025 criterion: hypertension if SBP ≥ 130 mmHg and/or DBP ≥ 80 mmHg, or self-reported lifetime prior diagnosis of arterial hypertension/high blood pressure by a health professional, considering that the 2025 AHA/ACC guideline replaces the 2017 guideline and retains the same adult BP categorization (12, 16).

Antihypertensive medication use was not included as an additional independent case-defining criterion because the ENDES medication item refers to receipt and/or purchase of medication to control blood pressure during the previous 12 months among participants with a prior diagnosis. Thus, medication use was operationalized as the treatment stage of the cascade and was nested within self-reported lifetime prior diagnosis rather than serving as an independent screening question for all respondents.

To quantify reclassification attributable to the lower diagnostic threshold proposed by U. S. guidelines, we defined the “ACC/AHA vs. ESH discordance zone” as the subgroup of participants without self-reported lifetime prior diagnosis who had SBP 130–139 mmHg and/or DBP 80–89 mmHg (i.e., values meeting ACC/AHA criteria but not reaching the ESH threshold of ≥140/90 mmHg).

#### 2.4.2 Blood pressure categories

Blood pressure was categorized using the average of the two measurements. When SBP and DBP fell into different categories, the category corresponding to the higher value was assigned.

According to the 2023 ESH classification, BP was categorized as optimal (SBP < 120 and DBP < 80 mmHg), normal (SBP 120–129 and/or DBP 80–84 mmHg), high-normal (SBP 130–139 and/or DBP 85–89 mmHg), and grade 1 hypertension (SBP 140–159 and/or DBP 90–99 mmHg), grade 2 (SBP 160–179 and/or DBP 100–109 mmHg), and grade 3 (SBP ≥ 180 and/or DBP ≥ 110 mmHg) (11). According to the 2025 ACC/AHA classification, BP was categorized as normal (SBP < 120 and DBP < 80 mmHg), elevated BP (SBP 120–129 and DBP < 80 mmHg), stage 1 hypertension (SBP 130–139 or DBP 80–89 mmHg), and stage 2 hypertension (SBP ≥ 140 or DBP ≥ 90 mmHg) (12). Additionally, to compare trends with prior literature, we defined prehypertension according to JNC7 (SBP 120–139 or DBP 80–89 mmHg) among participants without self-reported lifetime prior diagnosis (17).

#### 2.4.3 Hypertension care cascade

We constructed a hypertension care cascade based on population surveys, following previous approaches that quantify sequential losses from need for care to effective control (2, 4). The cascade included four stages: (i) hypertension (need for care), (ii) awareness of diagnosis, (iii) pharmacological treatment, and (iv) blood pressure control. Hypertension was defined according to the 2023 ESH and 2025 ACC/AHA criteria (11, 12).

Awareness of diagnosis was defined as the proportion of participants with hypertension who reported having ever been diagnosed in their lifetime with “arterial hypertension” or “high blood pressure” by a physician or other health professional. This corresponds to the ENDES lifetime prior diagnosis item, whereas pharmacological treatment was assessed for the previous 12 months. Responses of “does not know/does not remember” were treated as missing.

Pharmacological treatment was defined as the proportion of participants with a prior diagnosis who reported having received and/or purchased medications to control their BP in the last 12 months. Responses of “does not know/does not remember” were treated as missing.

BP control was defined as the proportion of participants receiving pharmacological treatment whose mean BP was below pre-specified thresholds. Two definitions were evaluated: conventional control (<140/90 mmHg) and strict control (<130/80 mmHg), consistent with thresholds commonly used in the literature and with contemporary recommendations (2, 11, 12).

For each stage we estimated: (a) conditional proportions, defined as the ratio of the number of participants in stage i to the number in stage i − 1, and (b) population proportions, defined as the ratio of the number of participants in stage i to the total number with hypertension.

#### 2.4.4 Blood pressure measurement

In ENDES, BP measurements were performed by trained field staff using a standardized home measurement protocol. According to ENDES technical documentation, participants remained seated with their back supported after a five-minute rest period, and the right arm was supported at heart level. Two complete measurements (SBP and DBP) were taken, separated by approximately two minutes, and the mean SBP and mean DBP were used for analysis.

#### 2.4.5 Independent and stratification variables

Stratification variables included age (18–29, 30–44, 45–59, 60–69, and ≥70 years), sex (women/men), area of residence (urban/rural), natural region (Metropolitan Lima, rest of the Coast, Highlands, and Jungle), and department (24 departments and the Constitutional Province of Callao).

Socioeconomic status was approximated using the household wealth index provided by ENDES, derived through principal component analysis of household assets and characteristics and categorized into population quintiles (Q1 = poorest; Q5 = richest).

Educational level was classified as primary or no education, secondary, and higher education according to the highest level attained. Health insurance was operationalized as a categorical variable (uninsured, Comprehensive Health Insurance [SIS], EsSalud, and other schemes—including Armed Forces/Police, EPS, and private insurance—), based on questionnaire self-report. Additionally, we included cardiovascular risk-related behaviors available in ENDES, measured by self-report: smoking status (never, former smoker, current smoker), alcohol consumption (none/never, non-excessive, excessive), and fruit and vegetable intake (≥5 servings/day: yes/no).

Body mass index (BMI) was calculated as weight (kg)/height (m)^2 from ENDES anthropometric measurements and included as a covariate (continuous or categorical, depending on the analysis). Survey year (2014–2024) was used as the time variable in trend analyses.

### 2.5 Statistical analysis

All analyses accounted for the complex ENDES sampling design (stratification, clustering, and weighting). Sampling weights, strata, and primary sampling units (PSUs) were declared, and standard errors were estimated using Taylor series linearization (svy commands). For strata with a single PSU, the singleunit(scaled) variance adjustment was applied. For analyses restricted to the analytic sample, subpopulation/domain estimation was used after retaining all records with valid design identifiers and positive weights. For pooled analyses across multiple years, PSU and stratum identifiers were made year-specific by concatenating survey year with the original codes to prevent collisions across cycles. Annual weights were normalized within each survey year and divided by the number of cycles included in each pooled period so that each annual survey contributed equally to pooled period estimates.

First, we conducted a descriptive analysis to characterize the study population. We reported absolute frequencies (unweighted) and weighted proportions with their corresponding 95% confidence intervals (CIs). Sociodemographic characteristics were tabulated for the entire period and for three *a priori* sub-periods: pre-ACC/AHA (2014–2016), post-ACC/AHA (2017–2019), and pandemic/post-pandemic (2020–2024). These sub-periods were selected to capture potential changes associated with publication of the 2017 ACC/AHA guidelines and with health restrictions related to the COVID-19 pandemic. Blood pressure was summarized using weighted means with standard errors for each survey year.

Second, we estimated the prevalence of hypertension for each survey year under both criteria (ESH and ACC/AHA), as well as the proportion in the diagnostic discordance zone (hypertension under ACC/AHA but not under ESH). For temporal comparability, we calculated age-standardized prevalences via direct standardization using the WHO World Standard Population as reference (18). Because the study included adults ≥18 years, we used an initial 18–19-year group and, from age 20 years onward, five-year age groups (20–24, 25–29, …, 75–79, and ≥80). Additionally, we estimated annual prevalences of prehypertension (JNC7), elevated BP (ACC/AHA), and high-normal BP (ESH). Geographic variability was described at the department level (25 geographic units) using an annual heatmap of the proportion with undiagnosed hypertension.

Third, the hypertension care cascade was constructed for each year from 2014 to 2024, reporting conditional and population proportions with 95% CIs, stratified by sex, area of residence, wealth quintile, age group, and insurance type. Sex gaps (women vs. men) and area gaps (urban vs. rural) were quantified as absolute differences in percentage points (pp) for the pooled 2021–2024 period, estimated using Wald contrasts adjusted for the sampling design and year-normalized pooled weights.

Fourth, temporal trends (2014–2024) were assessed using joinpoint segmented regression applied to 23 time series (three national series of age-standardized prevalence and 20 cascade series stratified by sex and area). For each annual indicator, we fitted segmented log-linear models within each segment j, weighted by the inverse of the variance of the annual estimator to reflect differences in precision across years YtlogYt=α+βj.t1E2 (19). Models with 0, 1, and 2 joinpoints were evaluated, imposing a minimum of three observations per segment to reduce overfitting. The number of joinpoints was selected using the Bayesian information criterion (BIC) (20). Statistical evidence of a change in slope was assessed using the Davies test (21). For each segment, the annual percent change (APC) and the average annual percent change (AAPC) were estimated. A positive AAPC indicates an average annual increase and a negative AAPC indicates a decrease.


$$\mathrm{APC}=[\exp(\beta_{j})-1]\;x\;100.$$

Fifth, socioeconomic inequalities at each cascade stage were evaluated using the Erreygers corrected concentration index (ECI), appropriate for bounded variables (0–1) such as prevalence or coverage indicators (22). The ECI was estimated for each cascade stage and each year from 2014 to 2024, using wealth quintile as the socioeconomic ranking variable and incorporating sampling weights. Standard errors and 95% CIs were obtained via robust estimation adjusted for clustering at the PSU level. Positive ECI values indicate concentration of the indicator among the richest quintiles (pro-rich inequality), whereas negative values indicate concentration among the poorest (pro-poor inequality).

Sixth, we projected national population-level indicators (awareness, population treatment, and population control <140/90 mmHg among ESH-defined hypertensive individuals) to 2030, assuming continuation of the observed log-linear trend. For each indicator, we extrapolated the 2014–2024 model using the AAPC as a constant rate of change. We derived 95% prediction intervals on the logarithmic scale and back-transformed them to percentages, constraining projections to the 0–100% range. Projections were compared with the global 80–80–80 target for 2030 (80% diagnosed, 80% treated among those diagnosed, and 80% controlled among those treated), equivalent to a minimum population control of 51.2% (0.8 × 0.8 × 0.8) among all individuals with hypertension $y_{t}=y_{2024}\times {\left(1+\frac{\text{AAPC}}{100}\right)}^{t-2024}\;$ (6). Gaps were expressed in percentage points between the projected value and the target.

Finally, the cascade in the last study year was translated into estimated absolute numbers of the national burden of undiagnosed and uncontrolled hypertension.

All analyses were conducted in Stata version 18.0 and R version 4.3. Joinpoint segmented regression was implemented in R using the segmented package. The ECI was computed in Stata using the user-written conindex command. Figures were generated in R using the ggplot2 and patchwork packages. Statistical significance was set at 0.05 (two-sided) for all hypothesis tests.

### 2.6 Ethical considerations

This study used exclusively publicly available microdata from ENDES downloaded from the INEI Microdata System. During fieldwork, ENDES includes informed consent procedures and an explicit confidentiality statement before the interview begins. For this analysis, we did not use identifiable information and did not contact participants; results are reported only in aggregated form. In accordance with ethical principles for research involving human participants, the study was conducted according to the Declaration of Helsinki. Given the public and de-identified nature of the data, new consent was not requested for this secondary analysis.

## 3 Results

### 3.1 Study population

We included 238,738 adults aged ≥18 years from 11 consecutive ENDES cycles (2014–2024) in the analytic subpopulation (Supplementary Figure S1). In the weighted sample, 51.5% were women and mean age was 42.5 years (95% CI: 42.4–42.6). Approximately six in ten participants were aged 18–44 years (18–29: 28.4%; 30–44: 30.6%), whereas those aged ≥70 years accounted for 8.7% (Table 1).

**Table 1:** Sociodemographic and clinical characteristics of the study population, ENDES 2014–2024.

Characteristics	Total	2014–2016	2017–2019	2020–2024
	(Pooled) (*n* = 238,738)	Pre-ACC/AHA (*n* = 77,229)	Post-ACC/AHA (*n* = 66,247)	Pandemic/post (*n* = 95,262)
Sex				
Women	133,405 (51.5)	42,596 (52.0)	37,252 (50.9)	53,557 (51.5)
Men	105,333 (48.5)	34,633 (48.0)	28,995 (49.1)	41,705 (48.5)
Age, mean (95% CI)	42.5 (42.4–42.6)	42.2 (42.0–42.4)	42.4 (42.2–42.6)	42.7 (42.6–42.9)
Age group, years				
18–29	69,531 (28.4)	21,937 (29.0)	19,658 (28.6)	27,936 (28.0)
30–44	87,311 (30.6)	27,389 (30.5)	24,241 (30.9)	35,681 (30.6)
45–59	44,058 (22.5)	15,036 (22.6)	11,955 (22.4)	17,067 (22.4)
60–69	20,294 (9.8)	6,674 (9.4)	5,540 (9.6)	8,080 (10.1)
70+	17,544 (8.7)	6,193 (8.5)	4,853 (8.5)	6,498 (8.9)
Area of residence				
Urban	159,506 (79.4)	50,440 (69.9)	45,482 (82.8)	63,584 (83.6)
Rural	79,232 (20.6)	26,789 (30.1)	20,765 (17.2)	31,678 (16.4)
Natural region				
Metropolitan Lima	29,315 (36.4)	8,906 (28.0)	8,360 (40.0)	12,049 (39.6)
Rest of the coast	69,974 (25.7)	22,471 (25.9)	19,841 (25.2)	27,662 (25.8)
Highlands	83,684 (25.3)	28,483 (31.8)	22,681 (23.3)	32,520 (22.4)
Jungle	55,765 (12.6)	17,369 (14.3)	15,365 (11.5)	23,031 (12.1)
Wealth quintile				
Poorest	69,129 (18.2)	21,233 (22.6)	18,877 (16.2)	29,019 (16.6)
Poor	60,227 (20.1)	19,334 (20.4)	16,889 (20.4)	24,004 (19.6)
Middle	46,288 (20.8)	15,071 (19.1)	12,918 (21.3)	18,299 (21.6)
Rich	36,076 (20.6)	12,059 (19.2)	10,009 (21.1)	14,008 (21.3)
Richest	27,018 (20.3)	9,532 (18.6)	7,554 (21.0)	9,932 (20.9)
Education level				
Primary/no education	49,739 (22.3)	16,600 (29.4)	12,303 (22.2)	20,836 (18.8)
Secondary	80,142 (43.1)	20,545 (41.2)	18,259 (41.7)	41,338 (44.7)
Higher education	57,112 (34.6)	14,005 (29.4)	13,490 (36.2)	29,617 (36.4)
Marital status				
Single	44,257 (28.4)	15,466 (30.3)	11,953 (28.0)	16,838 (27.3)
Married/cohabiting	101,675 (55.1)	33,077 (55.7)	28,655 (56.3)	39,943 (54.0)
Widowed/divorced/separated	29,377 (16.5)	9,225 (14.0)	7,587 (15.7)	12,565 (18.7)
Smoking status				
Never	195,724 (81.2)	61,729 (79.2)	54,214 (80.1)	79,781 (83.2)
Former smoker	18,060 (7.9)	6,402 (8.5)	5,055 (8.2)	6,603 (7.3)
Current smoker	24,949 (10.9)	9,093 (12.2)	6,978 (11.7)	8,878 (9.5)
Alcohol consumption				
No/never	159,015 (63.9)	51,133 (63.8)	44,056 (62.9)	63,826 (64.5)
Non-excessive	74,685 (33.5)	24,687 (34.0)	21,068 (34.9)	28,930 (32.2)
Excessive	5,038 (2.7)	1,409 (2.2)	1,123 (2.2)	2,506 (3.3)
Fruit/vegetable > = 5/day				
No	218,862 (91.0)	70,662 (91.1)	60,612 (90.5)	87,588 (91.3)
Yes	19,876 (9.0)	6,567 (8.9)	5,635 (9.5)	7,674 (8.7)
Self-reported diabetes				
No	230,703 (95.7)	75,011 (96.7)	64,203 (96.1)	91,489 (94.6)
Yes	7,856 (4.3)	2,174 (3.3)	1,993 (3.9)	3,689 (5.4)
BMI, mean (95% CI)	27.3 (27.2–27.3)	26.6 (26.6–26.7)	27.3 (27.2–27.3)	27.7 (27.6–27.7)
BMI category				
Underweight	2,531 (1.1)	920 (1.3)	659 (1.0)	952 (1.1)
Normal	85,150 (33.3)	30,777 (38.8)	23,476 (32.9)	30,897 (30.0)
Overweight	96,034 (40.6)	30,292 (39.4)	26,832 (41.2)	38,910 (41.0)
Obesity	55,023 (25.0)	15,240 (20.5)	15,280 (24.9)	24,503 (27.9)
SBP, mean (95% CI) mmHg	121.9 (121.8–122.0)	122.1 (121.9–122.3)	122.5 (122.3–122.8)	121.3 (121.1–121.5)
DBP, mean (95% CI) mmHg	73.3 (73.2–73.4)	71.5 (71.4–71.6)	72.4 (72.2–72.5)	75.2 (75.1–75.3)

Weighted using the complex survey design (svy) and year-normalized pooled weights for multi-year estimates. Continuous variables are reported as mean (95% CI) and categorical variables as unweighted n (weighted proportion, %). SBP, systolic blood pressure; DBP, diastolic blood pressure; BMI, body mass index; SIS, comprehensive health insurance; EsSalud, social health insurance.

Most participants lived in urban areas (79.4%), and 36.4% lived in Metropolitan Lima, followed by the rest of the Coast (25.7%), Highlands (25.3%), and Jungle (12.6%). Wealth quintiles were approximately evenly distributed (18.2–20.8% per quintile). Mean BMI was 27.3 kg/m^2 (95% CI: 27.2–27.3) and 65.6% had excess weight (overweight: 40.6%; obesity: 25.0%). Mean BMI increased from 26.6 kg/m^2 in 2014–2016 to 27.7 kg/m^2 in 2020–2024 (Table 1).

### 3.2 Blood pressure distribution

Weighted mean SBP was 123.1 mmHg in 2014, remained stable through 2021 (123.6 mmHg), and decreased to 119.5 mmHg in 2024. Median SBP decreased from 120 mmHg in 2014 to 115 mmHg in 2024. Mean DBP showed a different pattern: it remained between 72.1 and 72.4 mmHg in 2014–2019, increased to 77.0 mmHg in 2022, and was 75.9 mmHg in 2024. The 95th percentile of SBP decreased from 157.5 mmHg in 2014 to 148.0 mmHg in 2024, whereas the 95th percentile of DBP increased from 89.0 to 92.5 mmHg (Supplementary Figure S2).

### 3.3 Hypertension prevalence and BP categories

The weighted, non–age-standardized prevalence of hypertension under 2023 ESH criteria (≥140/90 mmHg or prior diagnosis) ranged from 18.6% (2016) to 23.9% (2021). Under 2025 ACC/AHA criteria (≥130/80 mmHg or prior diagnosis), prevalence ranged from 34.7% (2016) to 46.3% (2022). In 2024, prevalence was 22.2% (ESH) and 42.9% (ACC/AHA), with an absolute discordance between guidelines of 20.7 percentage points (Table 2).

**Table 2:** Hypertension prevalence and blood pressure categories according to ESH 2023 and ACC/AHA 2025 criteria, ENDES 2014–2024.

BP category	2014	2015	2016	2017	2018	2019	2020	2021	2022	2023	2024
Weighted proportions (%)											
ESH 2023 criteria (≥140/90 mmHg)											
Optimal (<120/80)	47.1	50.7	50.2	48.6	47.1	47.4	45.4	42.2	45.9	52.0	49.9
Normal (120–129/80–84)	22.2	22.5	22.3	22.6	22.7	22.5	22.0	23.0	21.9	20.6	20.5
High-normal (130–139/85–89)	14.8	13.8	14.0	14.3	14.6	15.4	15.3	16.2	14.9	13.7	14.4
Grade 1 (140–159/90–99)	11.3	9.4	9.3	10.4	11.4	10.7	12.8	13.3	12.4	10.2	11.3
Grade 2 (160–179/100–109)	3.1	2.2	3.0	2.9	3.0	2.9	3.0	3.8	3.5	2.5	2.8
Grade 3 (≥180/≥110)	1.5	1.3	1.1	1.2	1.3	1.2	1.4	1.6	1.3	1.0	1.2
Total HTN (≥140/90 or dx)	22.2	18.8	18.6	19.8	21.1	20.8	23.1	23.9	23.4	20.5	22.2
ACC/AHA 2025 criteria (≥130/80 mmHg)											
Normal (<120/80)	47.1	50.7	50.2	48.6	47.1	47.4	45.4	42.2	45.9	52.0	49.9
Elevated BP (120–129/<80)	18.0	18.5	18.5	18.4	17.1	17.7	16.6	15.1	11.5	13.2	11.3
Stage 1 (130–139/80–89)	19.0	17.9	17.8	18.4	20.2	20.2	20.7	24.0	25.4	21.1	23.6
Stage 2 (≥140/≥90)	15.9	13.0	13.5	14.6	15.6	14.8	17.3	18.7	17.2	13.7	15.2
Total HTN (≥130/80 or dx)	38.9	34.9	34.7	36.3	39.4	38.6	41.5	45.9	46.3	38.8	42.9
Guideline discordance											
Reclassified (discordance)	16.7	16.1	16.0	16.5	18.4	17.9	18.4	22.0	22.9	18.3	20.7

Weighted proportions using the complex survey design (svy). Blood pressure (BP) categories correspond to measured BP and are mutually exclusive. The total hypertension (HTN) row corresponds to crude prevalence defined as BP ≥140/90 mmHg or self-reported lifetime prior diagnosis (ESH 2023), and BP ≥130/80 mmHg or self-reported lifetime prior diagnosis (ACC/AHA 2025). Discordance (reclassified): individuals with hypertension under ACC/AHA but not under ESH (SBP 130–139 mmHg and/or DBP 80–89 mmHg without lifetime prior diagnosis).

**Figure 1:**
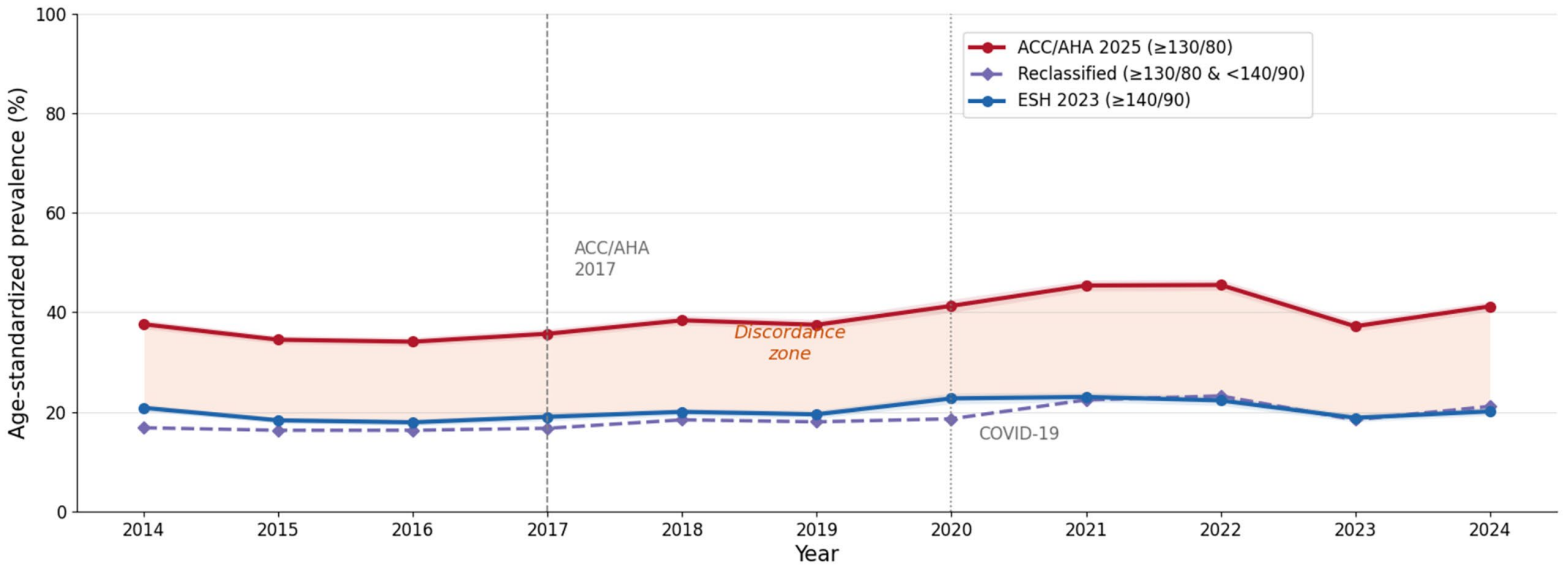
Age-standardized prevalence trends of hypertension and reclassified individuals according to ESH 2023 and ACC/AHA 2025. Age-standardization was performed using the WHO World Standard Population. ESH: Hypertension if SBP/DBP ≥ 140/90 mmHg or self-reported lifetime prior diagnosis. ACC/AHA: Hypertension if SBP/DBP ≥ 130/80 mmHg or self-reported lifetime prior diagnosis. Reclassified: Hypertension under ACC/AHA criteria but not under ESH criteria (SBP 130–139 mmHg and/or DBP 80–89 mmHg) among those without self-reported lifetime prior diagnosis.

After direct age-standardization to the WHO World Standard Population, ESH-defined hypertension prevalence ranged from 17.9% (95% CI: 17.2–18.5) in 2016 to 23.0% (95% CI: 22.0–24.0) in 2021; in 2024 it was 20.1% (95% CI: 19.3–20.9). For ACC/AHA-defined hypertension, values ranged from 34.1% (95% CI: 33.3–35.0) in 2016 to 45.5% (95% CI: 44.4–46.6) in 2022; in 2024 it was 41.2% (95% CI: 40.2–42.2). Age-standardized discordance (reclassified: 130–139/80–89 mmHg without prior diagnosis) ranged from 16.3% (95% CI: 15.6–17.0) in 2016 to 23.2% (95% CI: 22.3–24.2) in 2022; in 2024 it was 21.1% (95% CI: 20.2–22.0) (Supplementary Table S1; Figure 1).

Joinpoint regression identified no change points (0 joinpoints) in any series (Supplementary Table S5). The ESH-defined hypertension trend was stable (AAPC 0.88%; 95% CI: −0.78 to 2.57). In contrast, ACC/AHA-defined hypertension showed an increasing trend (AAPC 1.92%; 95% CI: 0.35 to 3.52), as did the reclassified group (AAPC 2.88%; 95% CI: 1.31 to 4.48) (Supplementary Table S5).

Regarding measured BP categories (independent of prior diagnosis), the proportion with BP < 120/80 mmHg was 47.1% in 2014, decreased to 42.2% in 2021, and increased to 49.9% in 2024. Under ESH, grade 1 hypertension (140–159/90–99) ranged from 9.3% (2016) to 13.3% (2021). Under ACC/AHA, elevated BP (120–129/<80) decreased from 18.5% (2015–2016) to 11.3% (2024), while stage 1 hypertension (130–139/80–89) reached 25.4% in 2022 (Table 2).

### 3.4 Hypertension care cascade

In 2014–2024, conditional cascade stages (diagnostic awareness among individuals with hypertension; pharmacological treatment among those reporting a diagnosis; and BP control among those reporting treatment) varied year-to-year without a sustained pattern of improvement. Among ESH-defined hypertensive individuals, awareness ranged from 44.0% (2021) to 54.8% (2023). Treatment among those diagnosed fluctuated between 57.0% (2014) and 65.3% (2020). Control <140/90 mmHg among those treated ranged from 42.8% (2021) to 59.0% (2024), whereas strict control <130/80 mmHg was consistently lower (26.6–34.3%). In 2024 (ESH), 53.6% of hypertensive individuals reported a prior diagnosis. Among them, 60.3% reported pharmacological treatment and, among those treated, 59.0% achieved BP < 140/90 mmHg. At the population level, this translated into 32.3% of hypertensive individuals being treated and 19.1% achieving BP < 140/90; strict control (<130/80) was 10.7%.

Under the 2025 ACC/AHA criterion, crude hypertension prevalence ranged from 34.7% (2016) to 46.3% (2022). Within this framework, diagnostic awareness among those meeting ACC/AHA criteria remained consistently below 30% throughout the period, ranging from 22.9% (2021) to 28.9% (2023) (Table 3).

**Table 3:** Hypertension care cascade, ENDES 2014–2024.

Stage	2014	2015	2016	2017	2018	2019	2020	2021	2022	2023	2024
Crude prevalence											
HTN ESH (≥140/90 or dx)	22.2	18.8	18.6	19.8	21.1	20.8	23.1	23.9	23.4	20.5	22.2
HTN ACC/AHA (≥130/80 or dx)	38.9	34.9	34.7	36.3	39.4	38.6	41.5	45.9	46.3	38.8	42.9
Care cascade (conditional proportions)											
Awareness (among ESH-defined hypertensives)	50.3	53.5	49.4	46.5	47.9	52.0	45.7	44.0	48.9	54.8	53.6
Awareness (among ACC/AHA-defined hypertensives)	28.7	28.8	26.6	25.3	25.6	28.0	25.4	22.9	24.7	28.9	27.7
Treatment (among diagnosed)	57.0	58.0	61.8	61.5	62.8	64.8	65.3	62.2	59.3	61.3	60.3
Control <140/90 (among treated)	48.8	50.6	51.4	51.6	46.9	52.8	52.3	42.8	51.0	56.1	59.0
Control <130/80 (among treated)	30.4	34.3	31.3	31.9	29.9	30.8	31.3	26.6	28.9	30.4	33.1
Population-level indicators (among all ESH-defined hypertensives)											
Overall treatment	28.7	31.0	30.5	28.6	30.1	33.7	29.8	27.3	29.0	33.6	32.3
Overall control <140/90	14.0	15.7	15.7	14.8	14.1	17.8	15.6	11.7	14.8	18.8	19.1
Overall control <130/80	8.7	10.6	9.5	9.1	9.0	10.4	9.3	7.3	8.4	10.2	10.7

Weighted proportions (%). Complex survey design.

Awareness = self-reported lifetime prior diagnosis among individuals with hypertension. Treatment = medication in the last 12 months among those diagnosed. Control = BP below threshold among those treated.

The largest loss point in 2024 was diagnosis: 46.4% of ESH-defined hypertensive individuals did not report a prior diagnosis. This gap was larger in men (58.8% undiagnosed) than in women (31.1%) and in adults aged 18–29 years (66.2%) (Supplementary Tables S2, S3).

### 3.5 Sex differences

During 2014–2024, hypertension prevalence defined by ESH was higher in men than in women. In 2024, ESH-defined hypertension prevalence was 25.3% in men and 19.3% in women [absolute difference: 6.0 percentage points (pp)]. When applying the 2025 ACC/AHA criteria, the prevalence gap widened (2024: 51.5% in men vs. 34.9% in women; difference: 16.6 pp).

Among individuals with ESH-defined hypertension, women had higher diagnostic awareness than men (2024: 68.9% vs. 41.2%; difference: 27.7 pp). Pharmacological treatment among those reporting a prior diagnosis was 62.6% in women and 57.2% in men (difference: 5.4 pp). Among those treated, control was higher in women for both the <140/90 mmHg threshold (60.6% vs. 56.6%; difference: 4.0 pp) and the <130/80 mmHg threshold (35.2% vs. 30.0%; difference: 5.2 pp).

The pattern of higher prevalence in men (under both ESH and ACC/AHA) and higher diagnostic awareness in women was consistent across all years; in contrast, gaps in treatment and control varied in magnitude across cycles (Supplementary Table S2).

**Figure 2:**
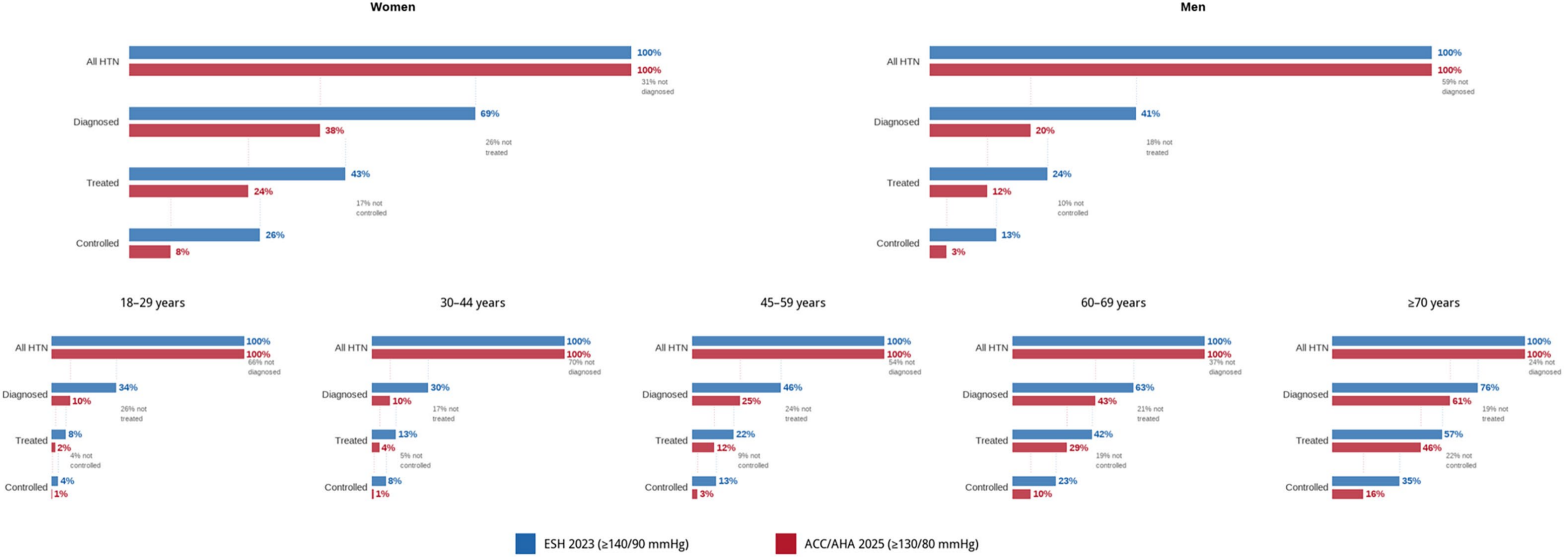
Hypertension care cascade, stratified by sex, age group, and diagnostic criterion. Gaps are expressed in percentage points (pp). Blue bars represent the ESH 2023 criterion, and red bars represent the ACC/AHA 2025 criterion.

Simultaneous stratification by sex and age group showed a marked gradient in the cascade (Figure 2). During 2014–2024, diagnostic awareness was higher in women than in men (68.9% vs. 41.2%) and increased with age (33.8% in 18–29 years vs. 77.1% in ≥70 years). Although control <140/90 mmHg among treated individuals remained in similar ranges for most subgroups (approximately 53–61% in 2024) (Supplementary Tables S2, S3), population-level performance remained limited by substantial losses at earlier stages (diagnosis and treatment initiation), especially under the ACC/AHA criterion due to the larger denominator of individuals classified as hypertensive (Figure 2).

### 3.6 Socioeconomic gradient

In 2024, ESH-defined hypertension prevalence was lower in the poorest quintile (Q1) than in the richest quintile (Q5): 17.5% vs. 29.1% (absolute difference: 11.6 pp). A similar pattern was observed under the 2025 ACC/AHA criteria (Q1: 37.5% vs. Q5: 51.5%; difference: 14.0 pp) (Supplementary Table S4).

Among individuals with ESH-defined hypertension, diagnostic awareness increased with wealth (2024: 47.4% in Q1 vs. 64.2% in Q5; difference: 16.8 pp). Among those reporting a prior diagnosis, pharmacological treatment was also higher in Q5 (48.6% in Q1 vs. 70.5% in Q5; difference: 21.9 pp). In contrast, BP control among those treated showed smaller differences for the <140/90 mmHg threshold (61.6% in Q1 vs. 64.8% in Q5) and did not show a consistent gradient for the <130/80 mmHg threshold (38.5% in Q1 vs. 34.0% in Q5).

The ECI for ESH-defined hypertension prevalence was positive in all years from 2014 to 2024, with a minimum in 2016 (ECI 0.045) and a maximum in 2022 (ECI 0.090), indicating concentration of hypertension among higher wealth quintiles. The ECI for treatment was larger than that for prevalence and awareness throughout the period (range 0.083–0.197), suggesting greater concentration of access to medication among the richest quintiles (Supplementary Figure S3).

### 3.7 Temporal trends in the stratified cascade

Joinpoint regression applied to 23 time series (three age-standardized prevalence series and 20 cascade indicators stratified by sex and area of residence) selected models with no change points (0 joinpoints) for all series. Accordingly, no statistically significant inflection points were identified, and trends were described as log-linear throughout 2014–2024 (Supplementary Table S5).

In the sex-stratified analysis, the only indicator with a statistically significant temporal change was strict control (<130/80 mmHg) among treated individuals: in women it showed a decreasing trend (AAPC −1.53%; 95% CI: −3.03 to −0.01), whereas in men it showed an increasing trend (AAPC +2.13%; 95% CI: 0.53 to 3.76). ESH-defined hypertension prevalence, diagnostic awareness, treatment, and conventional control (<140/90 mmHg) did not show significant changes in either sex.

In the analysis by area of residence, diagnostic awareness in rural areas was the only indicator with a significant trend (AAPC +1.17%; 95% CI: 0.02 to 2.34). In urban areas, no statistically significant trends were observed for any of the five indicators evaluated.

### 3.8 Cascade by health insurance type

In the pooled 2014–2024 period, analyses by insurance type showed substantial differences in the care cascade (Supplementary Figure S4). Under the 2023 ESH criterion, EsSalud affiliates had the highest hypertension prevalence (28.8%). Diagnostic awareness was higher in EsSalud (56.0%) and SIS (51.5%), whereas uninsured individuals had the lowest awareness (34.3%). Population-level treatment (overall treatment among individuals with hypertension) was higher in EsSalud (40.6%) and in the Armed Forces/EPS/private insurance group (36.4%). Overall control was low across all groups, particularly among the uninsured (7.7% under ESH and 2.3% under ACC/AHA). When applying the 2025 ACC/AHA criterion, hypertension prevalence increased due to the lower diagnostic threshold; however, proportions in subsequent stages (awareness, treatment, and control) decreased relative to ESH-based estimates.

### 3.9 Cascade gaps

Analysis of absolute gaps for 2021–2024 (Table 4) showed that the largest sex inequality was concentrated in diagnostic awareness: 66.2% in women versus 36.7% in men, corresponding to a men–women difference of −29.5 pp. In subsequent stages, sex gaps were smaller: treatment among those diagnosed 62.0% vs. 58.7% (−3.3 pp), control <140/90 among those treated 55.1% vs. 48.4% (−6.6 pp), and control <130/80 among those treated 31.9% vs. 26.6% (−5.3 pp). The urban–rural gap was smaller. Awareness was similar (49.9% urban vs. 51.2% rural; rural–urban: +1.3 pp). The main difference was observed in treatment among those diagnosed: 62.3% in urban vs. 50.8% in rural (rural–urban: −11.5 pp). In contrast, control among treated individuals was slightly higher in rural areas: control <140/90 52.1% vs. 55.1% (+3.0 pp) and control <130/80 29.3% vs. 34.6% (+5.3 pp).

**Table 4:** Absolute gaps in the hypertension cascade, ENDES 2021–2024.

Stage	Women (%)	Men (%)	Gap (pp)
Panel A: Sex gap, 2021–2024			
Awareness	66.2	36.7	−29.5
Treatment	62.0	58.7	−3.3
Control <140/90	55.1	48.4	−6.6
Control <130/80	31.9	26.6	−5.3

Stage	Urban (%)	Rural (%)	Gap (pp)
Panel B: Urban–rural gap, 2021–2024			
Awareness	49.9	51.2	+1.3
Treatment	62.3	50.8	−11.5
Control <140/90	52.1	55.1	+3.0
Control <130/80	29.3	34.6	+5.3

Gap (pp) = Men – Women / Rural – Urban. Estimates were calculated for the pooled 2021–2024 period using the complex survey design and year-normalized pooled weights. Awareness = self-reported lifetime prior diagnosis among individuals with hypertension (ESH 2023 definition). Treatment = medication in the last 12 months among those diagnosed. Control = BP below threshold among those treated. pp = percentage points.

### 3.10 Trends in prehypertensive stages

After direct age-standardization to the WHO World Standard Population, prehypertension prevalence according to JNC7 (SBP 120–139 or DBP 80–89 mmHg among individuals without prior diagnosis) remained around 33–34% between 2014 and 2020, peaked in 2021 (36.0%; 95% CI: 34.8–37.1), and decreased to 30.6% (95% CI: 29.7–31.6) in 2024. Elevated BP according to ACC/AHA (SBP 120–129 and DBP < 80 mmHg) decreased from 16.5% (95% CI: 15.8–17.2) in 2014 to 9.9% (95% CI: 9.3–10.5) in 2024. High-normal BP according to ESH (SBP 130–139 or DBP 85–89 mmHg) was relatively stable (12.8%; 95% CI: 12.2–13.5 in 2014), showed a transient increase in 2021 (14.7%; 95% CI: 13.8–15.5), and was 12.3% (95% CI: 11.5–13.0) in 2024 (Supplementary Table S6).

### 3.11 Geographic heterogeneity

Department-level analyses showed marked geographic heterogeneity in the proportion of undiagnosed hypertension (hypertensive individuals unaware of their condition), with year-to-year variation across the 25 geographic units during 2014–2024. In 2024, undiagnosed hypertension ranged from 14% in Ucayali to 86% in Ayacucho; persistently high values were observed in Cajamarca (84%) and Amazonas (82%), whereas the lowest were recorded in Lima (17%), San Martín (16%), and Lambayeque (19%) (Supplementary Figure S5).

### 3.12 Projection to 2030 and comparison with international targets

National trends in 2014–2024 for population-level hypertension care cascade indicators (ESH criterion) were consistent with a log-linear model with no change points; therefore, they were extrapolated to 2030 using the average annual percent change (AAPC, relative change). Estimated AAPCs were: diagnosis/awareness 0.21% per year, overall treatment 0.60% per year, and overall control 1.58% per year. In 2024, diagnosis/awareness among hypertensive individuals was 53.6%, overall treatment was 32.3%, and overall control (<140/90 mmHg) was 19.1%. Under this continuation scenario, projections for 2030 were: diagnosis/awareness 54.2%, overall treatment 33.5%, and overall control 21.0%.

When these projections were compared with the international 80–80–80 target for 2030 (80% of people with hypertension diagnosed, 80% of those diagnosed treated, and 80% of those treated with controlled blood pressure)—equivalent to population targets of 80% for diagnosis/awareness, 64% for overall treatment, and 51.2% for overall control—projected gaps for 2030 were 25.8, 30.5, and 30.2 percentage points, respectively.

## 4 Discussion

### 4.1 Key findings

This study provides the most extensive ENDES-based assessment to date to characterize the hypertension care cascade among Peruvian adults (2014–2024; n = 238,738). In 2024, for every 100 individuals with hypertension under 2023 ESH criteria (BP ≥ 140/90 mmHg or lifetime prior diagnosis), 53.6 knew their diagnosis; among those diagnosed, 60.3 received pharmacological treatment; and among those treated, 59.0 achieved control <140/90 mmHg, translating into an overall control of 19.1% (and 10.7% under the stricter <130/80 mmHg threshold). Accordingly, the largest bottleneck was detection (46.4% undiagnosed in 2024). Joinpoint regression identified no breakpoints (0 joinpoints) in the evaluated series, suggesting no abrupt changes in population-level trajectories over the period. Because ENDES is a repeated cross-sectional survey, these findings should be interpreted as annual population patterns rather than individual transitions through the cascade.

A second key finding is that using dual diagnostic criteria substantially changes the interpretation of trends. Whereas age-standardized hypertension prevalence under 2023 ESH criteria remained stable (AAPC 0.88%), prevalence under 2025 ACC/AHA criteria (BP ≥ 130/80 mmHg or prior diagnosis) increased (AAPC 1.92%) and, in parallel, the proportion of reclassified/discordant individuals (ACC/AHA-defined hypertension not meeting ESH criteria; AAPC 2.88%) also increased.

The divergent pattern observed for SBP and DBP also deserves consideration. Mean SBP and the upper SBP percentiles declined toward 2024, whereas mean DBP and the upper DBP tail increased. This may reflect a combination of mechanisms rather than a single epidemiological process. Lower SBP at the upper tail could be compatible with partial improvement in detection or treatment among individuals with more severe systolic hypertension, whereas increasing DBP may reflect changes in peripheral vascular resistance and cardiometabolic risk profiles, particularly the observed increase in BMI over the study period. Obesity-related hypertension is biologically plausible through renal, neurohumoral, sympathetic, and sodium-retention pathways; therefore, the concurrent increase in BMI provides a plausible explanatory context, although the repeated cross-sectional design precludes causal attribution. Measurement effects, survey-year variability, and the single-visit BP protocol should also be considered when interpreting this divergence.

Finally, no stratum approached the proposed 80–80–80 target for global hypertension control (80% diagnosed, 80% treated, and 80% controlled). In 2024, even the best-performing group (women) achieved only about 26% overall control <140/90 mmHg (≈13% in men), and among those aged ≥70 years—although awareness exceeded 75%—overall control was ~36%. Estimates of control among 18–29-year-olds were imprecise and were suppressed in several years due to insufficient sample sizes; nevertheless, low awareness (33.8% in 2024) indicates a critical detection gap. Consistently, socioeconomic inequalities widened along the cascade: the Erreygers concentration index showed more pronounced inequities in treatment than in awareness or prevalence, suggesting structural barriers to access and continuity of therapy.

### 4.2 Comparison with other studies

Our findings align with prior national evidence based on ENDES, but provide a broader comparative framework by integrating 11 consecutive rounds (2014–2024), simultaneously applying two current diagnostic criteria (ESH 2023 and ACC/AHA 2025), and evaluating trends using weighted log-linear (joinpoint) models. Overall, the pattern observed in Peru—low effective coverage of control and substantial losses in the transition “hypertensive → diagnosed → treated → controlled”—is consistent with reports from many low- and middle-income countries, where hypertension is characterized by a “detection deficit” followed by structural barriers to sustaining pharmacological treatment and follow-up (2, 4).

In Peru, Villarreal-Zegarra et al. analyzed ENDES 2015–2018 and reported an unfavorable pattern over a short interval: increases or stability in age-standardized prevalence accompanied by deterioration in awareness and control - (8). By extending analyses to 2024, our findings suggest that interpretations based on short windows can magnify transient fluctuations (e.g., changes in age composition, sampling variability, or service disruptions), whereas a decade-long perspective confirms that population-level cascade performance remains low. In particular, overall control <140/90 mmHg in 2024 (19.1% among ESH-defined hypertensive individuals) remains far from values observed in health systems with established hypertension control programs (23).

Carrillo-Larco et al. used six national surveys to describe mean blood pressure across cascade stages and also showed that the largest drop occurs before treatment—that is, at diagnosis and linkage to care - (9). This approach complements ours: whereas their analysis emphasizes shifts in mean BP by stage (useful for monitoring treatment intensity and control), our study adds (i) a formal assessment of temporal trends, (ii) quantification of control under conventional and strict thresholds (<140/90 and <130/80 mmHg), and (iii) systematic description of socioeconomic inequalities over time. The convergence of both approaches reinforces the interpretation that Peru’s gaps are not explained solely by the “quality of control” among treated individuals, but by accumulated losses at early stages, particularly timely diagnosis and sustained treatment initiation.

More recently, Diaz-Arocutipa et al. reported national trends from 2019 to 2023, a period that captures the pandemic and post-pandemic transition - (10). In the present study, the absence of detectable breakpoints (0 joinpoints) in the main series suggests that, even if episodic disruptions occurred, they did not abruptly modify the national trajectory when considering the full decade. This does not necessarily contradict findings from shorter windows, but highlights the value of longer series to infer structural health system performance.

In the international literature, a key source of comparative heterogeneity is the diagnostic threshold used. The 2017 ACC/AHA guideline lowered the cut-off to 130/80 mmHg to define hypertension and consequently increases prevalence and the size of the denominator over which cascade stages are calculated (16). Population impact studies in the United States showed that adopting the ACC/AHA threshold can reclassify a substantial proportion of adults as hypertensive and expand the universe eligible for preventive interventions, although the effect on pharmacological treatment indication depends on baseline cardiovascular risk (13). This phenomenon is not trivial: when the denominator grows (more people classified as hypertensive under a lower threshold), proportions of awareness, treatment, and control tend to decrease mechanically if the system does not expand diagnostic and treatment capacity at the same pace. The “dual-criterion” approach in our study makes this dynamic transparent and avoids misleading comparisons with studies that use a single threshold.

In Peru, the difference between definitions is substantial: in 2024, crude hypertension prevalence was 22.2% under ESH (≥140/90 mmHg or prior diagnosis) and 42.9% under ACC/AHA (≥130/80 mmHg or prior diagnosis), with a discordance zone close to one-fifth of the adult population. This pattern is consistent with international experiences where ACC/AHA reclassification concentrates in the 130–139/80–89 mmHg range (ACC/AHA stage 1 hypertension), a segment that represents an opportunity for primary prevention interventions (lifestyle, weight management, salt reduction) and, in high-risk subgroups, pharmacological treatment (11–13).

From a global perspective, NCD Risk Factor Collaboration (NCD-RisC) analyses documented heterogeneous advances in treatment and control between 1990 and 2019, with some countries reaching control levels above 50% and others showing minimal progress - (23). In parallel, the WHO has emphasized that uncontrolled hypertension continues to account for a very high mortality burden and that most people with hypertension worldwide still do not achieve control (3). Even considering differences in age range and standardization, the magnitude of population-level control observed in Peru remains below that of leading countries and is more consistent with the average performance of many low- and middle-income countries.

A particularly informative comparison comes from a multicountry evaluation of 44 low- and middle-income countries using individual-level microdata (1.1 million adults), which showed that the largest losses usually occur at the awareness (diagnosis) stage and that population-level effective control is low in most contexts (4). The Peruvian pattern is congruent with this evidence: even though control among treated individuals can approach 6 in 10 under the <140/90 threshold, overall control remains around 1 in 5 because a considerable fraction of hypertensive individuals are unaware of their condition and another fraction, even if diagnosed, does not initiate or sustain treatment. In this sense, our findings align with the idea that improving national control requires a chain of interventions (diagnosis + timely treatment + continuity + intensification) rather than isolated actions at a single link.

With respect to programmatic initiatives, the WHO HEARTS/Global Hearts package proposes protocol standardization, availability of essential medicines, monitoring systems, and strengthening of primary care; however, implementation evaluations in multiple countries show that even with adoption of the approach, initial population-level control is usually low and improves gradually as the program expands and matures (7, 24). This offers two lessons for Peru: first, the magnitude of the lag is not exceptional among countries without robust programs; and second, there is evidence that performance can improve substantially if large-scale strategies are implemented with continuity and monitoring, particularly when treatment regimens are simplified and clinical inertia is reduced (3, 6, 7, 24).

Compared with high-income countries, NHANES 2021–2023 data showed that approximately 59% of individuals with hypertension were aware of their diagnosis and about 51% received pharmacological treatment, with ~21% achieving control <130/80 mmHg among all individuals with hypertension (25). In Peru, overall control <130/80 in 2024 was 10.7% among ESH-defined hypertensive individuals, suggesting a substantial gap even under a strict threshold; moreover, the main differential does not seem to lie in control among treated individuals, but in earlier stages (awareness and overall treatment). In other words, comparison with NHANES suggests that to increase population-level control, Peru needs to increase timely diagnosis and treatment initiation/continuity in parallel, rather than focusing efforts solely on intensification among those already treated.

Regarding South Korea—one of the countries with the best historical performance in hypertension control—a national study (2009–2022) described sustained upward trends in management indicators, with signs of setback during the pandemic period and subsequent recovery (26). The absence of a clear pandemic break in our series may reflect differences in system resilience, but also limitations inherent to annual population surveys (e.g., reduced sample size in 2020, changes in measurement devices, or random variation). Nevertheless, contrast with South Korea underscores that achieving population-level control near or above 50% is feasible when continuous programs, effective coverage, and sustained availability of antihypertensive medications in primary care are in place.

The social inequalities observed in Peru also mirror international patterns. In many low- and middle-income countries, hypertension may be concentrated among higher-wealth groups (due to nutritional transition and obesity), while access to diagnosis and treatment is often even more concentrated among advantaged strata, widening inequities as the cascade progresses (4, 23). Use of the Erreygers concentration index—appropriate for bounded variables—allows more consistent comparison of the socioeconomic gradient across stages with different prevalences (22). Within this framework, the finding that inequality is greater in treatment than in awareness suggests that the critical barrier is not only detecting hypertension among poorer individuals, but ensuring sustained access to medications and clinical follow-up.

Finally, sex and age differences in the Peruvian cascade are consistent with global reports: women tend to have higher awareness and treatment than men, whereas young adults tend to exhibit lower diagnostic awareness, delaying entry into the cascade (4, 23). Nonetheless, the divergent trend in strict control by sex observed in our analysis (decrease in women and increase in men) warrants further exploration, as comparative evidence on <130/80 mmHg control by sex in population surveys is limited and could be influenced by differences in treatment intensity, adherence, comorbidities, and access to services (25). Overall, these comparisons position Peru as a country with intermediate-to-low performance in the region and a persistent structural gap relative to leading countries, reinforcing the urgency of systemic and sustained interventions.

### 4.3 Public health implications

Projections of the cascade to 2030 have direct implications for health policy: under the observed trajectory, Peru will not meet any of the 80–80–80 targets of the HEARTS initiative. For 2030, population-level control <140/90 mmHg is projected at 21.0% versus the required 51.2% (gap: 30.2 percentage points [pp]). Considering the value observed in 2024 (19.1%), closing the gap by 2030 would require an average absolute increase of approximately 5.3 pp. per year, implying an order-of-magnitude acceleration relative to the progress observed over the last decade. This deficit is clinically relevant: lowering blood pressure through pharmacological treatment is consistently associated with reductions in major cardiovascular events and mortality; therefore, sustained increases in population-level control have potentially large public health impact (27).

From a programmatic perspective, the results suggest that the main bottleneck is not only in the final control stage, but in the accumulated losses upstream. In 2024, out of every 100 ESH-defined hypertensive individuals, 53.6 knew their diagnosis, 32.3 received treatment (population indicator), and 19.1 achieved population-level control <140/90 mmHg. Accordingly, the largest marginal gains in population control would depend on expanding effective diagnosis and timely treatment initiation with continuity, in addition to optimizing therapeutic intensification. This pattern is consistent with multinational evidence from low- and middle-income countries, where low treatment coverage and discontinuity of care explain much of the low population-level control (4).

Young adults should be considered a priority group for early detection. In 2024, awareness among hypertensive adults aged 18–29 years remained low, indicating that most young adults with hypertension had not entered the cascade. This pattern is expected in a largely asymptomatic condition and may be amplified by lower routine health-care utilization in younger populations. From a life-course perspective, delayed detection in early adulthood can extend cumulative exposure to elevated BP and increase future cardiovascular risk. Opportunistic BP measurement in educational, occupational, community, and primary-care settings may therefore be necessary to reduce this early detection gap.

The finding of a significant trend limited to increased awareness in rural areas (stratified series) suggests that screening is reaching historically underserved territories, but is not translating into treatment or control. This decoupling is compatible with system-level constraints in primary care capacity, medicine supply, therapeutic continuity, follow-up, and retention and supports integrated interventions rather than isolated actions focused only on detection. Programmatic evidence indicates that intervention packages most likely to succeed combine systematic screening with standardized treatment protocols, validated measurement, performance monitoring, and strengthening of the supply chain (7, 24).

The socioeconomic inequalities observed along the cascade imply that universal strategies without an explicit equity component could widen gaps. Therefore, expanding coverage should be accompanied by measures to ensure financial protection and effective access: continuous availability of essential antihypertensive medicines without prohibitive co-payments, provision at the primary care level, and community strategies targeting populations with lower probability of diagnosis and treatment, with periodic monitoring of inequities as a performance indicator.

Differences by health insurance type suggest that socioeconomic inequality is reinforced by health-system fragmentation. EsSalud affiliates showed higher awareness and treatment than uninsured individuals, whereas the uninsured had the lowest overall control. This gradient is consistent with differential access to regular care, medication availability, and follow-up capacity across insurance schemes. Therefore, improving hypertension control in Peru requires more than expanding screening: it requires continuity of treatment and affordable access to essential antihypertensive medicines across SIS, EsSalud, and uninsured populations, with explicit monitoring of cascade indicators by insurance type.

Overall, our findings point to a national strategy aligned with HEARTS that prioritizes: (i) routine, standardized blood pressure measurement at all points of contact with the health system (including opportunistic and community screening); (ii) simplified, stepwise treatment protocols, favoring combination therapy when indicated to reduce therapeutic inertia and facilitate implementation; (iii) team-based care and task sharing with trained non-physician health workers under clinical supervision; (iv) assurance of continuous supply of essential medicines and validated measurement devices; and (v) information/registry systems with follow-up of cascade indicators and inequities by territory and socioeconomic status. Implementation of HEARTS/Global Hearts programs across multiple countries shows that substantial improvements in control are feasible when these actions are applied in an integrated manner (7, 24).

Finally, simultaneous consideration of diagnostic thresholds (ESH and ACC/AHA) raises a policy issue: adopting lower thresholds without increasing system capacity could expand the denominator of people requiring follow-up and potentially treatment, placing additional strain on primary care. In this context, strengthening population prevention and the capacity for protocolized management in primary care is a prerequisite for any normative reform that increases service demand.

### 4.4 Limitations

This study is based on repeated cross-sectional surveys and therefore describes annual population patterns; it cannot infer causality or individual trajectories within the cascade. Blood pressure was measured at a single visit (mean of two readings), which may introduce measurement error (e.g., white-coat effect/masked hypertension) relative to clinical confirmation across multiple occasions recommended by guidelines. In addition, awareness and treatment were based on self-report (potential differential error by socioeconomic status/territory), and ENDES does not capture adherence, medication type/dose, or number of drugs, limiting assessment of treatment quality and intensity beyond the binary treated/not treated indicator.

Additionally, the 2020 cycle had reduced sample size due to COVID-19, and the complete-case approach could have introduced selection bias; moreover, some subgroup or department-level estimates may be imprecise due to small effective sample sizes. Joinpoint analysis was applied to multiple series with only 11 annual points, which may limit power to detect subtle changes and requires caution regarding multiplicity. Inequality metrics are based on an asset-based wealth index and relative measures (ECI) that depend on the socioeconomic ranking within each year; therefore, temporal comparisons should be interpreted cautiously. Finally, 2030 projections assume continuation of trends and do not incorporate future changes in policy, supply, or external shocks; they should be considered informative planning scenarios rather than causal predictions.

## 5 Conclusion and recommendations

Across this series of national ENDES surveys (2014–2024), the population-level hypertension care cascade in Peru showed persistently low performance. In 2024, 53.6% of individuals with hypertension under the 2023 ESH criterion were aware of their condition, 32.3% received pharmacological treatment, and only 19.1% achieved population-level control <140/90 mmHg. Although control among treated individuals was 59.0%, the main bottlenecks occurred before treatment, reflecting substantial losses in diagnosis and therapeutic coverage. Comparing definitions showed a much larger burden under ACC/AHA 2025 than under ESH 2023 (42.9% vs. 22.2% in 2024) and an increasing discordance zone (SBP 130–139/DBP 80–89 mmHg without prior diagnosis), with direct implications for service planning and risk-based prevention strategies. If the observed trend continues, by 2030 awareness is projected at 54.2%, overall treatment at 33.5%, and overall control at 21.0%, leaving gaps of approximately 26–31 percentage points relative to the 80–80–80 targets (equivalent to 51.2% population-level control) promoted by the WHO HEARTS initiative.

We recommend implementing a national hypertension control strategy centered on primary care with an explicit equity focus: (i) systematic, repeated blood pressure screening at all primary care contact points and in community activities, using validated devices and quality assurance; (ii) simplified treatment protocols with timely, stepwise intensification, favoring combination therapy (ideally fixed-dose combinations when available) and task sharing with trained non-physician health teams in line with WHO guidance; (iii) continuous availability and affordability of essential antihypertensive medicines (including effective coverage for uninsured populations) to prevent expansion of screening from increasing inequities; and (iv) a monitoring and accountability system based on disaggregated cascade indicators (sex, age, area, quintile, and insurance) with subnational targets. Improving blood pressure coverage and control is a high-impact intervention to reduce cardiovascular disease and preventable mortality at the population level.

## Data Availability

The datasets analyzed in this study are publicly available from the INEI Microdata System (Sistema de Microdatos), including the ENDES 2014-2024 annual survey files: https://proyectos.inei.gob.pe/microdatos/. Further inquiries can be directed to the corresponding author.
