# Supplementary material for: Trends in the hypertension care cascade under dual guideline criteria in Peru, 2014–2024: a decade of nationally representative surveys with joinpoint analysis and projections toward the WHO 2030 targets
Source: Front Public Health. 2026 Jun 3;14:1830773. doi: 10.3389/fpubh.2026.1830773 (PMC13272369; doi:10.3389/fpubh.2026.1830773)
Supplement: Supplementary file 1 [file Supplementary_file_1.docx]

Supplementary Material

**
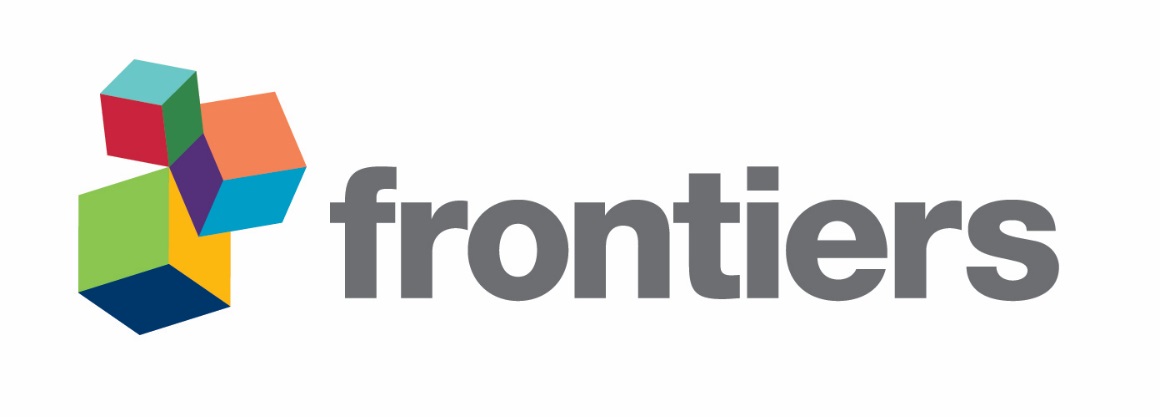
**

**Table S1.** Age-standardized prevalence of hypertension by diagnostic criterion, ENDES 2014–2024. Values are expressed as % (95% CI).

**Table S2.** Hypertension care cascade stratified by sex, ENDES 2014–2024

**Table S3.** Hypertension care cascade stratified by age group, ENDES 2014–2024.

**Table S4.** Hypertension care cascade stratified by wealth quintile, ENDES 2014–2024

**Table S5.** Joinpoint regression analysis of hypertension prevalence and care cascade indicators, ENDES 2014–2024.

**Table S6.** Age-standardized prevalence of prehypertension categories by year, ENDES 2014–2024. Values expressed as % (95% CI).

**Figure S1.** Flowchart of participant selection, ENDES 2014–2024.

**Figure S2.** Annual distribution of systolic blood pressure (SBP) (A) and diastolic blood pressure (DBP) (B), ENDES 2014–2024. Lines represent the 5th, 25th, 50th, 75th, and 95th percentiles.

**Figure S3.** Socioeconomic inequalities in hypertension prevalence and the care cascade under the 2023 ESH criterion, ENDES 2014–2024. ECI: Erreygers corrected concentration index. Positive values indicate concentration among higher wealth quintiles (pro-rich inequality).

**Figure S4**. Hypertension care cascade by health insurance type (pooled 2014–2024), under ESH 2023 and ACC/AHA 2025 criteria. Prevalence, awareness, population-level treatment, and population-level control proportions (%) are shown; treatment and control are expressed among all individuals with hypertension within each insurance group.

**Figure S5**. Proportion of undiagnosed hypertension by department, ENDES 2014–2024. Weighted proportions using the complex survey design (svy).

**Supplementary Text 1.** STROBE checklist (cross-sectional studies).

Supplementary Material

Table S1. Age-standardized prevalence of hypertension by diagnostic criterion, ENDES 2014–2024. Values are expressed as % (95% CI).

|  | **2014** | **2015** | **2016** | **2017** | **2018** | **2019** | **2020** | **2021** | **2022** | **2023** | **2024** |
| --- | --- | --- | --- | --- | --- | --- | --- | --- | --- | --- | --- |
| **HTA – ESH (≥140/90 o dx)** | 20.8 (20.1–21.5) | 18.3 (17.7–18.9) | 17.9 (17.2–18.5) | 19.0 (18.2–19.8) | 20.0 (19.2–20.7) | 19.5 (18.7–20.3) | 22.7 (21.6–23.8) | 23.0 (22.0–24.0) | 22.3 (21.3–23.2) | 18.8 (18.0–19.6) | 20.1 (19.3–20.9) |
| **HTA – ACC/AHA (≥130/80 o dx)** | 37.6 (36.7–38.5) | 34.5 (33.7–35.3) | 34.1 (33.3–35.0) | 35.7 (34.8–36.7) | 38.4 (37.5–39.4) | 37.5 (36.5–38.6) | 41.3 (40.0–42.6) | 45.4 (44.3–46.5) | 45.5 (44.4–46.6) | 37.2 (36.2–38.3) | 41.2 (40.2–42.2) |
| **Reclassified (discordance)** | 16.8 (16.1–17.5) | 16.3 (15.6–16.9) | 16.3 (15.6–17.0) | 16.7 (15.9–17.5) | 18.4 (17.6–19.3) | 18.0 (17.2–18.9) | 18.6 (17.5–19.7) | 22.4 (21.4–23.4) | 23.2 (22.3–24.2) | 18.4 (17.6–19.3) | 21.1 (20.2–22.0) |

*ESH: hypertension if SBP/DBP ≥140/90 mmHg or self-reported lifetime prior diagnosis. ACC/AHA: hypertension if SBP/DBP ≥130/80 mmHg or self-reported lifetime prior diagnosis. Reclassified: hypertension under ACC/AHA criteria but not under ESH criteria (SBP 130–139 mmHg and/or DBP 80–89 mmHg) among those without self-reported lifetime prior diagnosis. Age-standardization was performed using the WHO World Standard Population. dx: lifetime prior diagnosis.*

**Table S2.** Hypertension care cascade stratified by sex, ENDES 2014–2024

|  | **2014** | **2015** | **2016** | **2017** | **2018** | **2019** | **2020** | **2021** | **2022** | **2023** | **2024** |
| --- | --- | --- | --- | --- | --- | --- | --- | --- | --- | --- | --- |
| **Women** | | | | | | | | | | | |
| **HTN prev ESH (%)** | **20.0** | **18.1** | **17.1** | **17.9** | **18.7** | **18.6** | **20.5** | **20.8** | **20.7** | **18.7** | **19.3** |
| **HTN prev ACC/AHA (%)** | **31.4** | **28.0** | **27.6** | **28.3** | **30.7** | **30.2** | **33.2** | **37.4** | **37.5** | **31.0** | **34.9** |
| Awareness (%) | 64.1 | 67.9 | 63.2 | 63.6 | 66.5 | 66.4 | 62.3 | 61.7 | 64.5 | 70.1 | 68.9 |
| Treatment (%) | 58.4 | 59.3 | 61.5 | 63.8 | 63.9 | 65.3 | 62.3 | 60.2 | 63.1 | 62.4 | 62.6 |
| Control <140/90 (%) | 51.8 | 55.5 | 56.1 | 52.8 | 50.6 | 54.4 | 61.0 | 47.2 | 51.8 | 59.6 | 60.6 |
| Control <130/80 (%) | 35.9 | 39.5 | 36.8 | 35.6 | 33.9 | 34.5 | 38.0 | 28.5 | 30.9 | 32.6 | 35.2 |
| **Men** | | | | | | | | | | | |
| **HTN prev ESH (%)** | **24.7** | **19.5** | **20.2** | **21.8** | **23.5** | **23.0** | **25.8** | **27.1** | **26.3** | **22.4** | **25.3** |
| **HTN prev ACC/AHA (%)** | **47.5** | **42.2** | **42.1** | **44.7** | **48.5** | **47.2** | **50.3** | **54.9** | **55.6** | **47.2** | **51.5** |
| Awareness (%) | 37.3 | 39.4 | 37.2 | 31.7 | 32.6 | 40.1 | 31.9 | 29.6 | 35.9 | 41.0 | 41.2 |
| Treatment (%) | 54.9 | 55.9 | 62.1 | 57.6 | 60.9 | 64.2 | 70.1 | 65.5 | 53.7 | 59.6 | 57.2 |
| Control <140/90 (%) | 43.6 | 41.9 | 44.4 | 49.4 | 40.3 | 50.6 | 39.8 | 36.0 | 49.7 | 50.4 | 56.6 |
| Control <130/80 (%) | 20.9 | 24.9 | 23.1 | 24.9 | 22.9 | 25.6 | 21.7 | 23.7 | 25.4 | 27.0 | 30.0 |

*Weighted proportions (%). Complex survey design accounted for.*

Table S3. Hypertension care cascade stratified by age group, ENDES 2014–2024.

|  | **2014** | **2015** | **2016** | **2017** | **2018** | **2019** | **2020** | **2021** | **2022** | **2023** | **2024** |
| --- | --- | --- | --- | --- | --- | --- | --- | --- | --- | --- | --- |
| **18–29 years** |  |  |  |  |  |  |  |  |  |  |  |
| HTN prev ESH (%) | 6.8 | 6.8 | 7.4 | 9.2 | 10.2 | 6.7 | 8.9 | 8.1 | 9.9 | 5.2 | 7.2 |
| HTN prev AHA (%) | 22.2 | 30.5 | 30.1 | 34.3 | 35.7 | 21.7 | 27.8 | 28.6 | 29.4 | 23.0 | 24.5 |
| Awareness (%) | 27.0 | 14.7 | 10.7 | 10.7 | 17.1 | 31.4 | 26.6 | 23.2 | 35.1 | 31.1 | 33.8 |
| Treatment (%) | 16.3 | 16.1 | 47.4 | 24.4 | 50.6 | 37.2 | 30.4 | 22.6 | 25.2 | 19.0 | 23.0 |
| *Control <140/90 (%)* | *—* | *—* | *—* | *—* | *—* | *—* | *—* | *—* | *—* | *—* | *—* |
| *Control <130/80 (%)* | *—* | *—* | *—* | *—* | *—* | *—* | *—* | *—* | *—* | *—* | *—* |
| **30–44 years** |  |  |  |  |  |  |  |  |  |  |  |
| HTN prev ESH (%) | 13.7 | 11.8 | 12.1 | 13.3 | 16.5 | 12.4 | 12.7 | 16.1 | 15.8 | 10.2 | 12.3 |
| HTN prev AHA (%) | 32.3 | 37.8 | 38.1 | 38.0 | 44.4 | 32.6 | 32.5 | 43.0 | 43.8 | 30.3 | 37.1 |
| Awareness (%) | 44.4 | 33.8 | 26.6 | 21.5 | 22.9 | 34.4 | 25.4 | 26.7 | 28.0 | 31.3 | 31.6 |
| Treatment (%) | 32.1 | 24.4 | 32.7 | 29.8 | 51.2 | 48.3 | 36.8 | 34.5 | 30.6 | 29.3 | 46.2 |
| Control <140/90 (%) | 56.1 | 41.4 | 74.7 | 71.4 | 60.7 | 50.9 | 65.3 | 52.8 | 51.7 | 89.3 | 53.7 |
| Control <130/80 (%) | 42.5 | 31.6 | 44.2 | 38.1 | 40.6 | 29.0 | 38.1 | 27.8 | 33.7 | 39.8 | 24.5 |
| **45–59 years** |  |  |  |  |  |  |  |  |  |  |  |
| HTN prev ESH (%) | 29.0 | 27.1 | 27.5 | 29.0 | 28.8 | 25.5 | 32.9 | 30.6 | 31.9 | 27.6 | 29.3 |
| HTN prev AHA (%) | 48.6 | 46.5 | 48.7 | 49.1 | 52.7 | 47.1 | 53.3 | 55.8 | 59.8 | 49.7 | 54.2 |
| Awareness (%) | 50.7 | 50.0 | 48.5 | 40.1 | 43.1 | 50.7 | 43.3 | 41.0 | 44.1 | 49.5 | 46.5 |
| Treatment (%) | 52.6 | 57.3 | 51.7 | 61.6 | 53.3 | 57.7 | 65.4 | 54.2 | 55.7 | 50.4 | 48.0 |
| Control <140/90 (%) | 61.3 | 57.7 | 51.9 | 62.7 | 60.9 | 61.3 | 65.7 | 47.0 | 53.5 | 55.9 | 59.2 |
| Control <130/80 (%) | 36.3 | 41.1 | 28.0 | 38.4 | 35.1 | 45.6 | 35.7 | 27.2 | 26.0 | 29.9 | 27.3 |
| **60–69 years** |  |  |  |  |  |  |  |  |  |  |  |
| HTN prev ESH (%) | 47.9 | 42.9 | 42.7 | 42.2 | 45.1 | 45.7 | 49.0 | 48.8 | 46.1 | 44.7 | 41.6 |
| HTN prev AHA (%) | 62.2 | 58.8 | 58.8 | 59.0 | 62.6 | 65.4 | 66.8 | 69.0 | 67.1 | 62.0 | 60.8 |
| Awareness (%) | 54.0 | 57.8 | 56.4 | 49.9 | 58.7 | 61.0 | 51.4 | 48.3 | 62.6 | 62.3 | 62.7 |
| Treatment (%) | 67.7 | 69.5 | 67.9 | 65.1 | 72.5 | 76.3 | 70.6 | 73.2 | 64.3 | 66.3 | 64.7 |
| Control <140/90 (%) | 51.8 | 48.7 | 48.3 | 51.5 | 40.8 | 52.2 | 53.0 | 36.9 | 52.1 | 60.8 | 53.2 |
| Control <130/80 (%) | 31.6 | 27.8 | 27.7 | 36.7 | 25.8 | 33.0 | 28.3 | 22.1 | 26.4 | 30.6 | 33.4 |
| **≥70 years** |  |  |  |  |  |  |  |  |  |  |  |
| HTN prev ESH (%) | 60.9 | 61.3 | 59.8 | 62.1 | 59.8 | 61.9 | 66.3 | 61.6 | 59.5 | 56.0 | 56.5 |
| HTN prev AHA (%) | 72.7 | 72.2 | 72.1 | 73.6 | 75.6 | 75.8 | 77.5 | 73.0 | 74.8 | 70.8 | 70.4 |
| Awareness (%) | 60.3 | 61.6 | 61.8 | 61.6 | 63.3 | 64.9 | 68.9 | 65.2 | 67.6 | 73.7 | 77.1 |
| Treatment (%) | 78.8 | 80.6 | 83.2 | 80.6 | 78.9 | 79.4 | 86.6 | 81.3 | 81.0 | 79.4 | 77.1 |
| Control <140/90 (%) | 37.8 | 38.8 | 42.1 | 40.4 | 39.4 | 44.3 | 40.2 | 34.9 | 48.8 | 46.9 | 61.3 |
| Control <130/80 (%) | 21.7 | 24.7 | 25.1 | 20.8 | 24.5 | 19.4 | 28.8 | 20.4 | 31.6 | 31.0 | 34.5 |

*Weighted proportions (%). Complex survey design accounted for. ESH: European Society of Hypertension 2023 (≥140/90 mmHg or lifetime prior diagnosis); AHA: American College of Cardiology/American Heart Association 2025 (≥130/80 mmHg or lifetime prior diagnosis). Awareness: proportion of ESH-hypertensives aware of diagnosis. Treatment: proportion of diagnosed receiving antihypertensive medication. Control: proportion of treated with BP below threshold.*

*— Estimates for control among treated in the 18–29 age group were suppressed due to insufficient unweighted sample size (n < 30 treated individuals in most survey years), which produced unstable estimates with unacceptably wide confidence intervals.*

Table S4. Hypertension care cascade stratified by wealth quintile, ENDES 2014–2024

|  | **2014** | **2015** | **2016** | **2017** | **2018** | **2019** | **2020** | **2021** | **2022** | **2023** | **2024** |
| --- | --- | --- | --- | --- | --- | --- | --- | --- | --- | --- | --- |
| **Q1 (Poorest)** | | | | | | | | | | | |
| HTN prev ESH (%) | 19.1 | 19.6 | 22.6 | 23.7 | 21.1 | 18.4 | 19.3 | 17.9 | 20.5 | 18.3 | 17.5 |
| HTN prev AHA (%) | 36.5 | 39.5 | 42.0 | 42.8 | 43.6 | 36.7 | 37.8 | 38.8 | 41.8 | 38.4 | 37.5 |
| Awareness (%) | 43.0 | 35.5 | 41.5 | 40.8 | 43.1 | 47.1 | 42.2 | 41.5 | 55.0 | 55.1 | 47.4 |
| Treatment (%) | 41.8 | 46.0 | 44.5 | 52.7 | 49.4 | 48.2 | 52.6 | 51.6 | 41.4 | 49.6 | 48.6 |
| Control <140/90 (%) | 71.4 | 42.1 | 54.2 | 37.4 | 51.4 | 54.4 | 57.1 | 50.8 | 54.7 | 59.4 | 61.6 |
| Control <130/80 (%) | 49.1 | 28.2 | 35.0 | 26.6 | 29.0 | 32.2 | 22.9 | 30.3 | 31.5 | 36.0 | 38.5 |
| **Q2 (Poor)** | | | | | | | | | | | |
| HTN prev ESH (%) | 20.0 | 23.1 | 21.9 | 20.6 | 27.1 | 20.3 | 19.6 | 22.5 | 19.7 | 19.7 | 21.0 |
| HTN prev AHA (%) | 35.8 | 42.9 | 43.5 | 40.8 | 52.0 | 36.5 | 41.7 | 43.2 | 43.9 | 40.4 | 41.0 |
| Awareness (%) | 48.1 | 47.4 | 49.8 | 43.4 | 45.3 | 46.2 | 45.2 | 38.5 | 42.2 | 52.8 | 50.1 |
| Treatment (%) | 44.5 | 59.1 | 62.8 | 65.2 | 55.5 | 59.7 | 53.1 | 58.8 | 50.2 | 50.7 | 53.0 |
| Control <140/90 (%) | 53.9 | 50.3 | 45.1 | 49.5 | 38.8 | 47.4 | 49.9 | 51.6 | 48.4 | 61.3 | 50.8 |
| Control <130/80 (%) | 35.2 | 32.9 | 29.4 | 30.2 | 27.2 | 34.9 | 38.3 | 34.5 | 19.1 | 29.1 | 28.8 |
| **Q3 (Middle)** | | | | | | | | | | | |
| HTN prev ESH (%) | 21.6 | 23.4 | 25.8 | 25.8 | 29.4 | 21.6 | 23.2 | 25.8 | 26.9 | 21.1 | 22.4 |
| HTN prev AHA (%) | 38.2 | 44.0 | 46.3 | 47.3 | 52.6 | 41.9 | 42.5 | 46.7 | 49.0 | 40.2 | 42.4 |
| Awareness (%) | 48.2 | 49.3 | 49.9 | 44.2 | 46.5 | 49.8 | 42.5 | 46.7 | 43.8 | 53.0 | 51.7 |
| Treatment (%) | 48.1 | 64.0 | 63.8 | 62.9 | 62.4 | 61.3 | 58.9 | 61.4 | 55.7 | 63.1 | 63.1 |
| Control <140/90 (%) | 46.4 | 38.5 | 44.1 | 58.6 | 41.5 | 47.5 | 56.3 | 39.3 | 52.8 | 54.6 | 53.2 |
| Control <130/80 (%) | 27.8 | 21.1 | 24.8 | 29.8 | 25.8 | 27.6 | 31.1 | 27.3 | 39.8 | 28.4 | 33.6 |
| **Q4 (Rich)** | | | | | | | | | | | |
| HTN prev ESH (%) | 26.0 | 30.7 | 29.7 | 30.6 | 30.3 | 22.1 | 26.5 | 25.6 | 25.1 | 23.4 | 25.8 |
| HTN prev AHA (%) | 42.5 | 51.4 | 50.6 | 52.1 | 53.8 | 41.4 | 44.9 | 50.8 | 50.0 | 43.0 | 47.9 |
| Awareness (%) | 54.1 | 60.5 | 49.1 | 41.4 | 50.7 | 53.4 | 42.5 | 39.1 | 50.1 | 56.5 | 54.0 |
| Treatment (%) | 62.7 | 73.3 | 67.3 | 66.1 | 74.2 | 70.8 | 63.9 | 59.6 | 64.7 | 63.6 | 58.6 |
| Control <140/90 (%) | 47.3 | 45.1 | 50.3 | 49.9 | 47.4 | 45.3 | 56.1 | 45.4 | 51.7 | 56.3 | 54.5 |
| Control <130/80 (%) | 30.9 | 30.1 | 31.7 | 37.8 | 25.2 | 27.8 | 24.4 | 23.7 | 32.6 | 34.8 | 26.7 |
| **Q5 (Richest)** | | | | | | | | | | | |
| HTN prev ESH (%) | 25.4 | 30.6 | 31.2 | 33.5 | 33.1 | 26.7 | 31.1 | 30.0 | 32.6 | 25.1 | 29.1 |
| HTN prev AHA (%) | 43.8 | 51.2 | 52.5 | 53.7 | 54.3 | 44.6 | 47.4 | 54.1 | 57.8 | 42.1 | 51.5 |
| Awareness (%) | 53.3 | 58.4 | 56.5 | 48.6 | 50.0 | 60.2 | 53.4 | 48.9 | 53.3 | 56.4 | 64.2 |
| Treatment (%) | 73.6 | 71.5 | 81.3 | 77.4 | 79.6 | 79.0 | 88.1 | 74.4 | 72.8 | 71.2 | 70.5 |
| Control <140/90 (%) | 49.2 | 52.8 | 47.0 | 49.4 | 47.1 | 58.7 | 49.4 | 32.5 | 50.0 | 50.6 | 64.8 |
| Control <130/80 (%) | 27.0 | 34.4 | 22.9 | 25.6 | 31.3 | 32.6 | 34.0 | 17.0 | 23.6 | 29.5 | 34.0 |

*Weighted proportions (%). Q1 = poorest, Q5 = richest.*

Table S5. Joinpoint regression analysis of hypertension prevalence and care cascade indicators, ENDES 2014–2024.

| **Series** | **Joinpoints** | **AAPC (%)** | **95% CI** | **p** | **Trend** |
| --- | --- | --- | --- | --- | --- |
| ***Age-standardized prevalence*** | | | | | |
| Hypertension prevalence—ESH | 0 | 0.88 | −0.78 to 2.57 | 0.261 | Stable |
| Hypertension prevalence—ACC/AHA | 0 | **1.92** | 0.35 to 3.52 | 0.022 | **Ascending *** |
| Reclassified (discordance) | 0 | **2.88** | 1.31 to 4.48 | 0.002 | **Ascending *** |
| ***Care cascade by area of residence — Urban*** | | | | | |
| Hypertension prevalence—ESH | 0 | 1.13 | −0.52 to 2.81 | 0.156 | Stable |
| Awareness | 0 | −0.19 | −1.83 to 1.47 | 0.801 | Stable |
| Treatment | 0 | 0.16 | −0.62 to 0.95 | 0.656 | Stable |
| Control <140/90 | 0 | 1.32 | −0.37 to 3.04 | 0.113 | Stable |
| Control <130/80 | 0 | 0.04 | −1.57 to 1.68 | 0.957 | Stable |
| ***Care cascade by area of residence — Rural*** | | | | | |
| Hypertension prevalence—ESH | 0 | 0.51 | −0.60 to 1.63 | 0.326 | Stable |
| Awareness | 0 | **1.17** | 0.02 to 2.34 | 0.047 | **Ascending *** |
| Treatment | 0 | 1.05 | −0.59 to 2.71 | 0.182 | Stable |
| Control <140/90 | 0 | 0.34 | −1.06 to 1.75 | 0.597 | Stable |
| Control <130/80 | 0 | −2.03 | −4.69 to 0.71 | 0.126 | Stable |
| ***Care cascade by sex — Women*** | | | | | |
| Hypertension prevalence—ESH | 0 | 0.71 | −0.58 to 2.02 | 0.246 | Stable |
| Awareness | 0 | 0.35 | −0.47 to 1.18 | 0.360 | Stable |
| Treatment | 0 | 0.59 | −0.07 to 1.25 | 0.073 | Stable |
| Control <140/90 | 0 | 0.79 | −0.66 to 2.26 | 0.251 | Stable |
| Control <130/80 | 0 | **−1.53** | −3.03 to −0.01 | 0.049 | **Descending *** |
| ***Care cascade by sex — Men*** | | | | | |
| Hypertension prevalence—ESH | 0 | 1.87 | −0.19 to 3.98 | 0.071 | Stable |
| Awareness | 0 | 0.11 | −2.38 to 2.66 | 0.923 | Stable |
| Treatment | 0 | 0.60 | −1.08 to 2.31 | 0.443 | Stable |
| Control <140/90 | 0 | 1.60 | −0.97 to 4.24 | 0.195 | Stable |
| Control <130/80 | 0 | **2.13** | 0.53 to 3.76 | 0.015 | **Ascending *** |

AAPC: average annual percent change; CI: confidence interval. Weighted log-linear models of the form ln(rate) = α + β·year were fitted, where APC = (exp(β) − 1) × 100. Weights were inversely proportional to the variance of each estimate. Models with 0, 1, and 2 joinpoints were evaluated; the best-fitting model was selected by the Bayesian Information Criterion (BIC), and the significance of candidate joinpoints was assessed using the Davies test. The 95% CI for the AAPC was calculated using the t-distribution with n − 2 degrees of freedom (df = 9). No joinpoints were identified in any series, indicating linear trends throughout 2014–2024.

*** p < 0.05.**

Table S6. Age-standardized prevalence of prehypertension categories by year, ENDES 2014–2024. Values expressed as % (95% CI).

| **Classification** | **2014** | **2015** | **2016** | **2017** | **2018** | **2019** | **2020** | **2021** | **2022** | **2023** | **2024** |
| --- | --- | --- | --- | --- | --- | --- | --- | --- | --- | --- | --- |
| Prehypertension (JNC7: 120–139/80–89) | 33.2 (32.3–34.1) | 32.9 (32.1–33.7) | 33.2 (32.4–34.1) | 33.7 (32.7–34.7) | 33.9 (32.9–34.8) | 33.9 (32.9–35.0) | 33.7 (32.4–35.0) | 36.0 (34.8–37.1) | 33.2 (32.1–34.3) | 30.1 (29.0–31.1) | 30.6 (29.7–31.6) |
| Elevated BP (ACC/AHA: 120–129/<80) | 16.5 (15.8–17.2) | 16.8 (16.1–17.4) | 17.2 (16.5–17.9) | 17.2 (16.4–18.0) | 15.5 (14.7–16.2) | 16.1 (15.3–16.9) | 15.3 (14.3–16.2) | 13.9 (13.1–14.8) | 10.3 (9.6–11.0) | 11.8 (11.0–12.5) | 9.9 (9.3–10.5) |
| High-normal (ESH: 130–139/85–89) | 12.8 (12.2–13.5) | 12.3 (11.7–12.9) | 12.4 (11.8–13.1) | 12.6 (11.8–13.3) | 13.0 (12.3–13.7) | 13.4 (12.6–14.2) | 13.4 (12.5–14.3) | 14.7 (13.8–15.5) | 13.2 (12.4–14.0) | 11.5 (10.8–12.2) | 12.3 (11.5–13.0) |

*Age-standardized using the WHO World Standard Population. JNC7: Seventh Report of the Joint National Committee on Prevention, Detection, Evaluation, and Treatment of High Blood Pressure. ACC/AHA: American College of Cardiology/American Heart Association 2025. ESH: European Society of Hypertension 2023. Prehypertension (JNC7) = SBP 120–139 or DBP 80–89 mmHg without lifetime prior diagnosis. Elevated BP (ACC/AHA) = SBP 120–129 and DBP <80 mmHg. High-normal (ESH) = SBP 130–139 or DBP 85–89 mmHg.*

Figure S1. Flowchart of participant selection, ENDES 2014–2024.


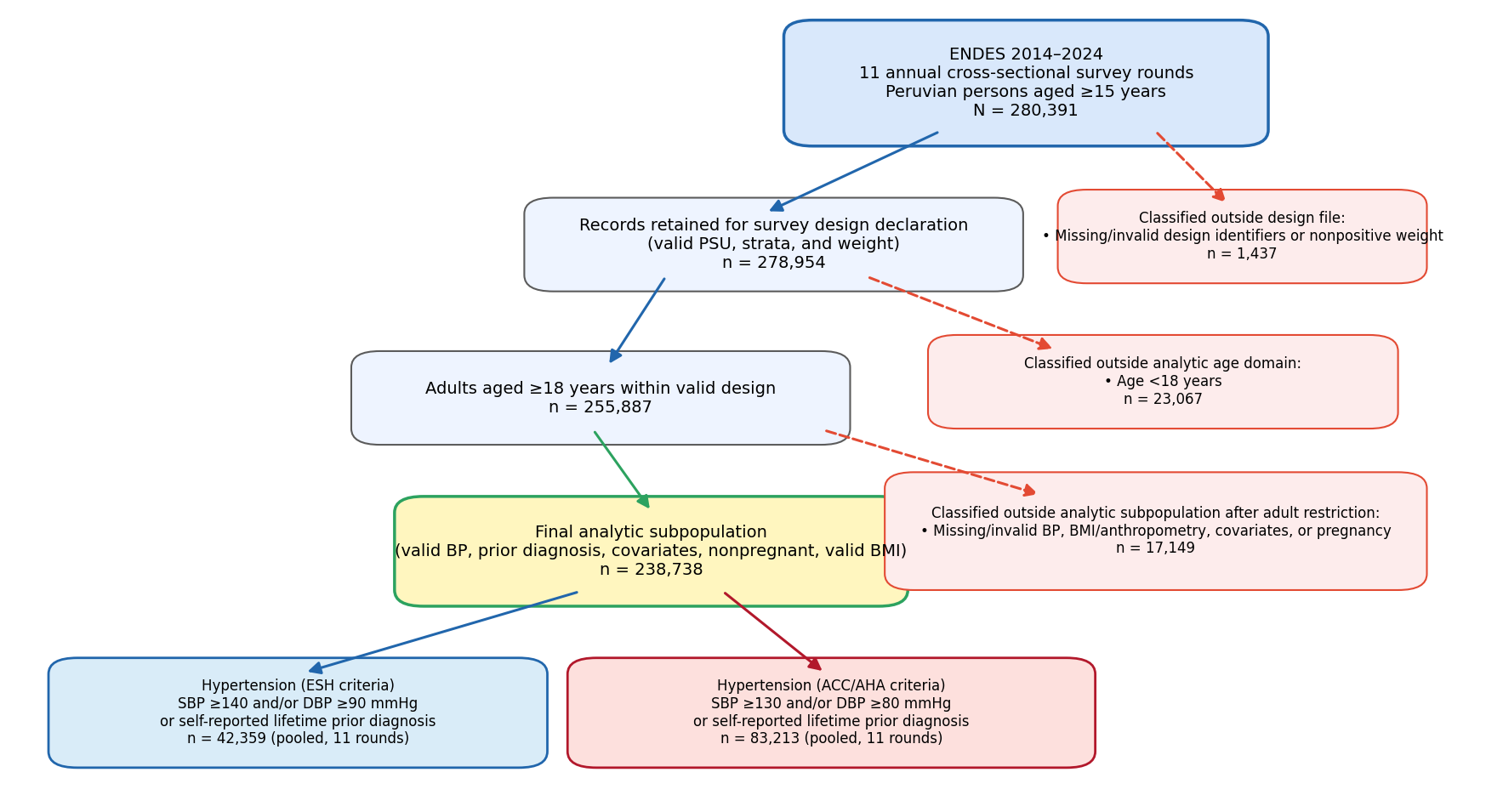


Figure S2. Annual distribution of systolic blood pressure (SBP) (A) and diastolic blood pressure (DBP) (B), ENDES 2014–2024. Lines represent the 5th, 25th, 50th, 75th, and 95th percentiles.

**
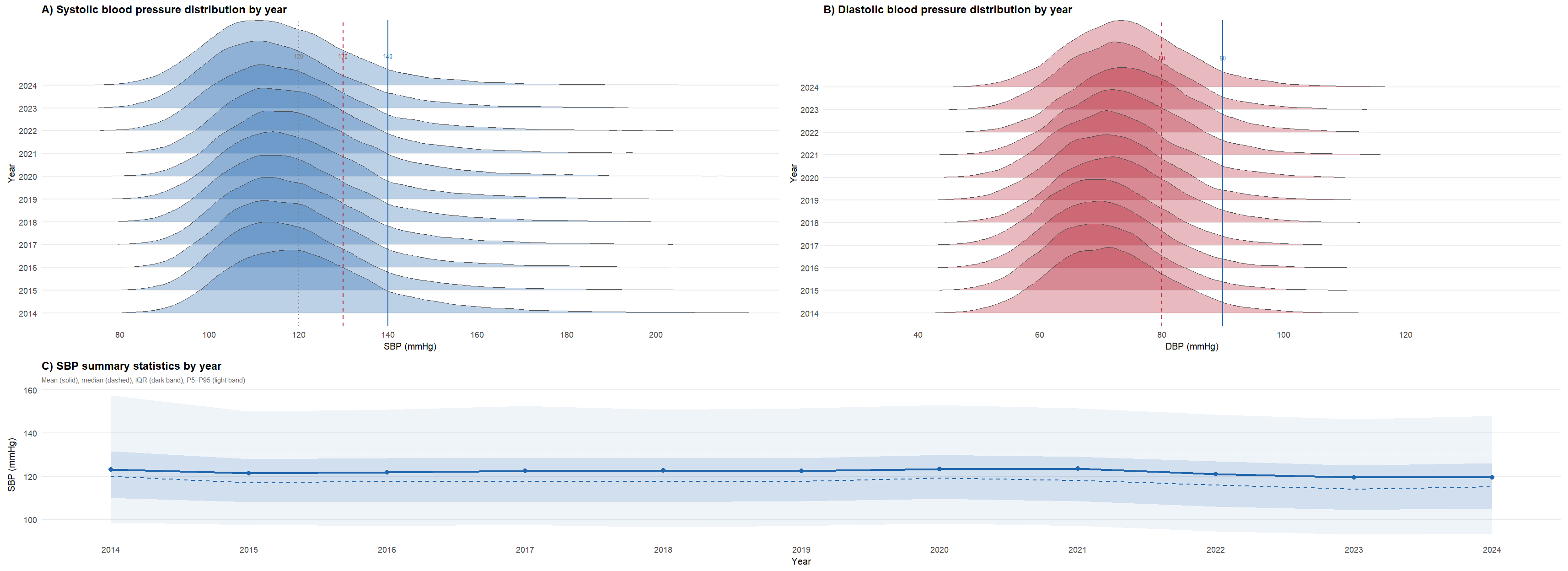
**

Figure S3. Socioeconomic inequalities in hypertension prevalence and the care cascade under the 2023 ESH criterion, ENDES 2014–2024. ECI: Erreygers corrected concentration index. Positive values indicate concentration among higher wealth quintiles (pro-rich inequality).

**
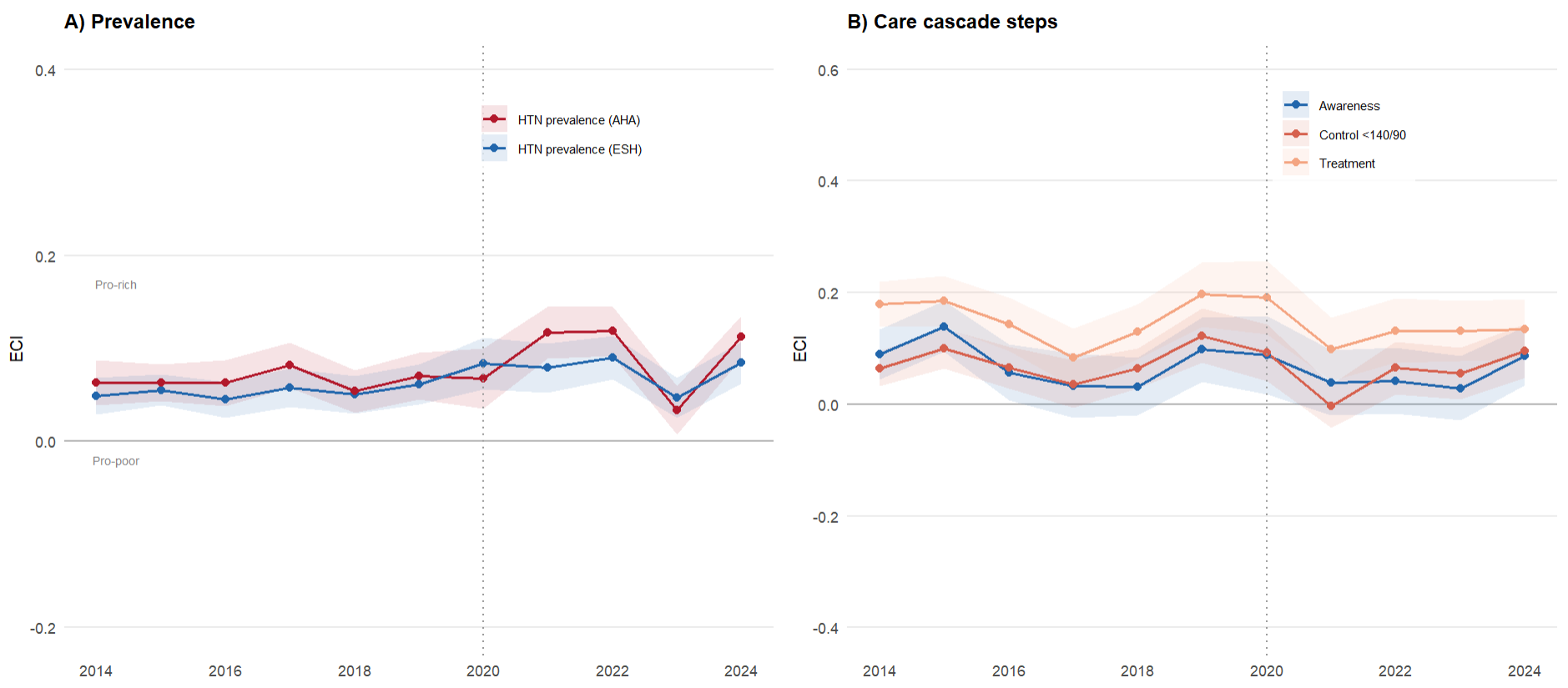
**

Figure S4. Hypertension care cascade by health insurance type (pooled 2014–2024), under ESH 2023 and ACC/AHA 2025 criteria. Prevalence, awareness, population-level treatment, and population-level control proportions (%) are shown; treatment and control are expressed among all individuals with hypertension within each insurance group.


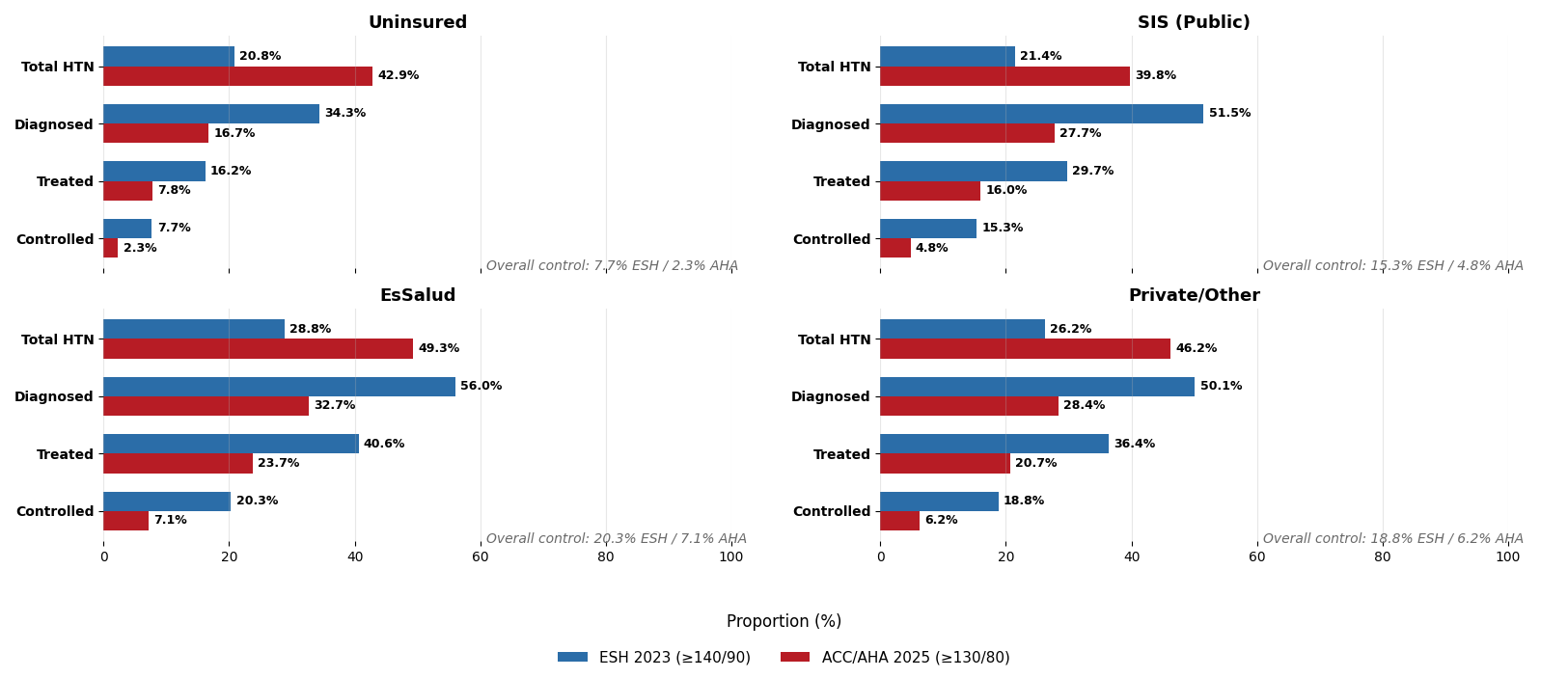


Figure S5. Proportion of undiagnosed hypertension by department, ENDES 2014–2024. Weighted proportions using the complex survey design (svy).
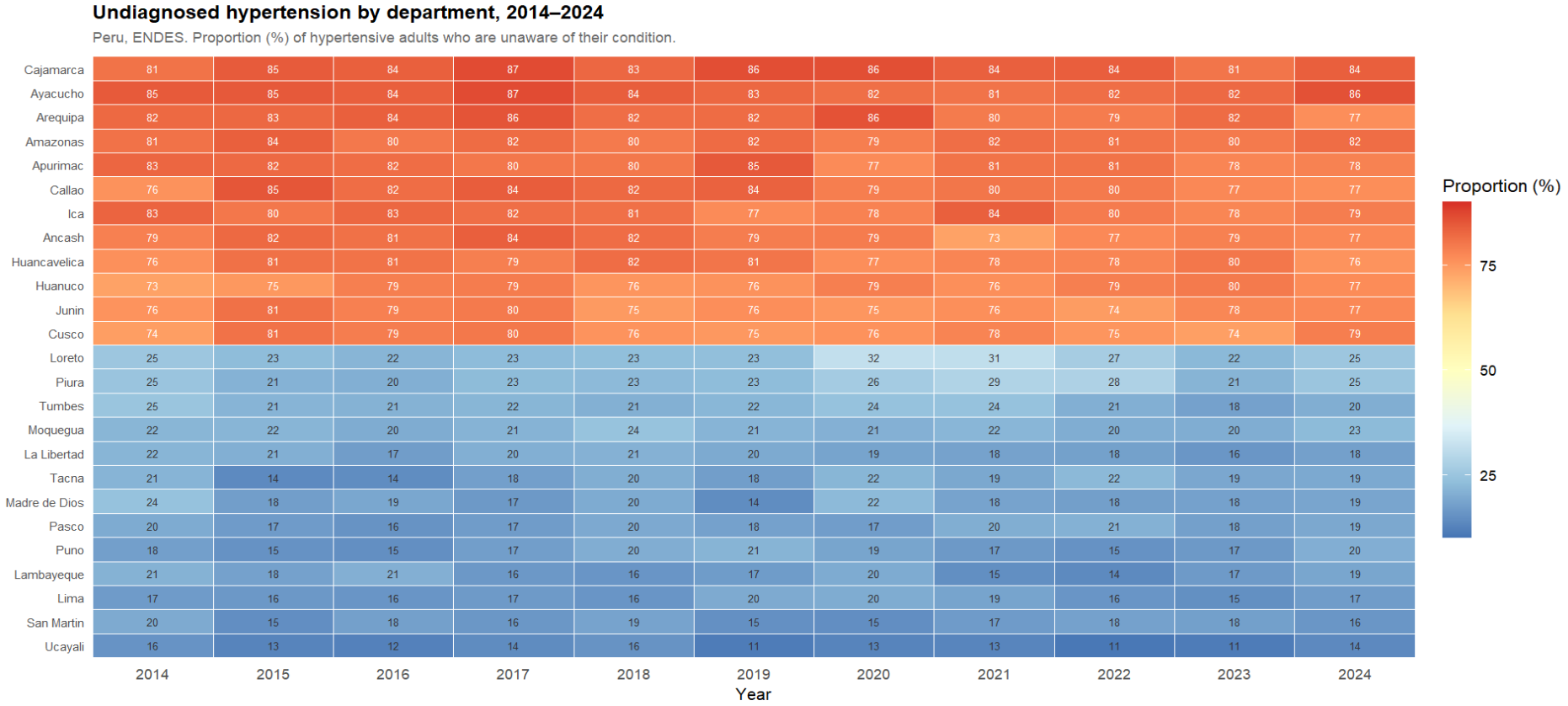


Supplementary Material 1. STROBE checklist (cross-sectional studies).

| **No.** | **Recommendation** | **Location in the manuscript** |
| --- | --- | --- |
| **TITLE AND ABSTRACT** | | |
| **1** | **(a) Indicate the study’s design with a commonly used term in the title or the abstract. (b) Provide in the abstract an informative and balanced summary of what was done and what was found.** | (a) Title: 'a decade of nationally representative surveys with joinpoint analysis and projections toward the WHO 2030 targets'. Abstract (Methods): repeated cross-sectional analysis; Methods: analytical cross-sectional observational study. (b) Abstract includes background (prior gaps), methods (238,738 adults; ENDES 2014–2024; dual ESH/ACC/AHA criteria; joinpoint; Erreygers), results (prevalence AAPC; 2024 cascade with specific figures; inequalities; 2030 projections with ~30 pp gaps vs 80–80–80), and conclusions. |
| **INTRODUCTION** | | |
| **2** | Background/rationale: Explain the scientific background and rationale for the investigation being reported. | Introduction, paragraphs 1–3: global hypertension burden (GBD 2019, NCD-RisC), attributable mortality (WHO 2023), the care cascade concept, the WHO HEARTS initiative with 80–80–80 targets for 2030, estimates of avoidable deaths (Nature Medicine), and limitations of prior studies in Peru (Villarreal-Zegarra 2015–2018, Carrillo-Larco 2015–2020, Diaz-Arocutipa 2019–2023): short periods, a single diagnostic threshold, and no projections or concentration indices. |
| **3** | Objectives: State specific objectives, including any prespecified hypotheses. | Introduction, last paragraph: four explicit objectives: (a) describe 2014–2024 trends in the cascade under dual criteria (ESH 2023 and ACC/AHA 2025); (b) identify change points using joinpoint regression; (c) quantify socioeconomic inequalities with the Erreygers index; and (d) project the cascade to 2030 and compare it with WHO HEARTS targets. No prespecified hypotheses were formulated. |
| **METHODS** | | |
| **4** | Study design: Present key elements of study design early in the paper. | Methods, “Study design”: analytical cross-sectional observational study using public-access secondary data from ENDES 2014–2024 (11 consecutive years). STROBE guidelines followed (Supplementary Material 1). |
| **5** | Setting: Describe the setting, locations, and relevant dates, including periods of recruitment, exposure, follow-up, and data collection. | Methods, “Data source”: ENDES is led by the National Institute of Statistics and Informatics (INEI), a national survey with a complex, stratified, two-stage probabilistic sampling design. It is representative at the national level, by area (urban/rural), by natural region, and by department (24 departments + the Constitutional Province of Callao). Analyzed period: 11 annual cycles (2014–2024). Methods, “Statistical analysis” (descriptive component): sub-periods pre-ACC/AHA (2014–2016), post-ACC/AHA (2017–2019), and pandemic/post-pandemic (2020–2024). |
| **6** | (a) Participants: Give the eligibility criteria, and the sources and methods of selection of participants. (b) For case-control studies: Give the matching criteria and the number of controls per case. | (a) Methods, “Data source” and “Population and sample”: usual resident adults selected for the ENDES Health Questionnaire, restricted to ≥18 years. Inclusion: ≥2 recorded SBP/DBP measurements and valid prior hypertension diagnosis information to define awareness. Classified outside the analytic subpopulation: implausible SBP/DBP values (SBP <70 or >270 mmHg; DBP <30 or >150 mmHg) or inconsistent measurements (SBP ≤ DBP); records with incomplete sampling design identifiers; missing data in key stratification covariates (sex, age, urban/rural area, natural region, wealth quintile); pregnancy; and missing valid weight/height for BMI. Figure S1 (Results/Supplementary material): flowchart with analytic subpopulation restrictions. (b) Not applicable (repeated cross-sectional study). |
| **7** | Variables: Clearly define all outcomes, exposures, predictors, potential confounders, and effect modifiers. Give diagnostic criteria, if applicable. | Methods, “Variables”: Hypertension defined according to ESH 2023 (SBP ≥140 mmHg and/or DBP ≥90 mmHg, or self-reported lifetime prior diagnosis) and ACC/AHA 2025 (SBP ≥130 mmHg and/or DBP ≥80 mmHg, or self-reported lifetime prior diagnosis). ACC/AHA vs ESH discordance zone: SBP 130–139 mmHg and/or DBP 80–89 mmHg among individuals without lifetime prior diagnosis. BP categories according to ESH 2023 and ACC/AHA 2025; JNC7 prehypertension for comparability. Cascade: (i) hypertension, (ii) awareness (self-reported lifetime prior diagnosis among hypertensive individuals), (iii) pharmacological treatment (received and/or purchased antihypertensive medication in the last 12 months among those diagnosed), and (iv) control (<140/90 and <130/80 mmHg among those treated), with conditional and population proportions. Stratification/covariates: age (groups), sex, area, natural region, department, wealth quintile, education, insurance, behaviors (tobacco/alcohol/fruit-vegetable intake), BMI, and year. |
| **8** | Data sources/measurement: For each variable of interest, give sources of data and details of methods of assessment (measurement). | Methods, “Blood pressure measurement”: BP measured by trained staff using a standardized home protocol (5-minute seated rest, back supported, right arm at heart level), two measurements (SBP and DBP) separated by ~2 minutes; the mean of both readings was used. Methods, “Variables”: awareness and treatment via self-report (operational definitions); BMI from measured weight and height; wealth index provided by ENDES. |
| **9** | Bias: Describe any efforts to address potential sources of bias. | Methods: incorporation of the complex sampling design to produce representative estimates; direct age standardization (WHO World Standard Population) for temporal comparability; cleaning/excluding implausible or inconsistent BP measurements; “don’t know/don’t remember” treated as missing. Discussion, “Limitations”: potential self-report bias (diagnosis and treatment), BP measured in a single visit (mean of two readings), reduced 2020 sample size and complete-case analytic subpopulation; no information on adherence/type/dose/number of drugs; limited power for joinpoint (11 annual points) and continuity assumptions for 2030 projections. |
| **10** | Study size: Explain how the study size was arrived at. | Methods and Results: all eligible participants from 11 ENDES cycles (2014–2024) were included. Analytic sample: 238,738 adults (after eligibility criteria; Figure S1). No a priori sample size calculation was performed; study size was determined by survey data availability. |
| **11** | Quantitative variables: Explain how quantitative variables were handled in the analyses. If applicable, describe which groupings were chosen and why. | Methods, “Variables”: BP as the mean of two measurements for definition/categorization; age in 5 groups for stratification (18–29, 30–44, 45–59, 60–69, ≥70) and in five-year age groups for direct age standardization; wealth quintile in 5 categories; BMI as continuous and categorical.  Results, “Blood pressure distribution” and Figure S2: SBP/DBP described with weighted means and, additionally, median and percentiles by year. |
| **12** | **(a) Statistical methods: Describe all statistical methods, including those used to control for confounding. (b) Describe any methods used to examine subgroups and interactions. (c) Explain how missing data were addressed. (d) Cross-sectional studies: If applicable, describe analytical methods taking account of sampling strategy. (e) Describe any sensitivity analyses.** | (a) Methods, “Statistical analysis” (six components): (1) Descriptive analysis with weighted frequencies by three sub-periods; (2) Crude and age-standardized prevalence (direct method, WHO World Standard Population) under ESH and ACC/AHA; (3) Conditional and population care cascade with 95% CIs, stratified by sex, area, wealth quintile, age, and insurance; (4) Joinpoint regression (23 series: 3 prevalence + 10 by sex + 10 by area), weighted log-linear models, APC, AAPC, Davies test, selection by BIC; (5) Erreygers index by stage and year; (6) Linear projections to 2030 with 95% prediction intervals. Department-level heatmap of undiagnosed hypertension. Software: Stata 18.0 (svy) and R 4.3. (b) Methods: cascade stratified by sex (Table S2; Figure 2), age group (Table S3), wealth quintile (Table S4), area of residence (Table S5), and insurance type (Figure S4). Sex and area gaps quantified as pp differences for 2021–2024 (Table 4). Joinpoint regression applied separately to urban, rural, women, and men. (c) Methods, “Population and sample”: classification of participants outside the analytic subpopulation with missing data in main stratification variables and with inconsistencies in sampling design variables. Flowchart: Figure S1 details analytic subpopulation restrictions. Complete-case analysis. (d) Methods, “Statistical analysis”: all analyses used Stata svy commands incorporating sampling weights, PSUs, and strata. Single-unit scaled method for strata with a single PSU. (e) Limitations: sensitivity analyses excluding 2020 (COVID-19 sample reduction) and using a single BP measurement (instead of the mean of two), both consistent with the main analysis. |
| **RESULTS** | | |
| **13** | **(a) Participants: Report numbers of individuals at each stage of the study. (b) Give reasons for non-participation at each stage. (c) Consider use of a flow diagram.** | (a) Results, “Study population”: 238,738 adults ≥18 years included after applying eligibility criteria across 11 ENDES cycles (2014–2024). Figure S1: flowchart with sequential analytic subpopulation restrictions. (b) Figure S1: detailed reasons for exclusion (implausible BP values, missing key stratification variables, sampling design inconsistencies). (c) Figure S1: participant selection flow diagram. |
| **14** | (a) Descriptive data: Give characteristics of study participants. (b) Indicate number of participants with missing data for each variable of interest. | (a) Results, “Study population” and Table 1: sociodemographic and clinical characteristics for the full period and by sub-periods; Results, “Blood pressure distribution” and Figure S2/Supplementary tables: annual SBP/DBP summaries. (b) Figure S1: overall analytic subpopulation restrictions by criteria (including missing key covariates). |
| **15** | Outcome data: Cross-sectional studies: Report numbers of outcome events or summary measures. | Results: crude prevalence in 2024—ESH: 22.2%; ACC/AHA: 42.9%; discordance zone: 20.7% (Table 2). Age-standardized prevalences by year (Figure 1). 2024 cascade: awareness 53.6%, treatment among diagnosed 60.3%, control <140/90 among treated 59.0%, overall control 19.1% (Table 3). |
| **16** | Main results: Give unadjusted estimates and, if applicable, confounder-adjusted estimates and their precision (e.g., 95% confidence interval). | Results and Table S5: joinpoint trends with no change points; AAPC (95% CI) for age-standardized prevalence—Hypertension ESH 0.88% (95% CI: −0.78 to 2.57), Hypertension ACC/AHA 1.92% (95% CI: 0.35 to 3.52), and reclassified 2.88% (95% CI: 1.31 to 4.48). Results and tables: crude prevalences by year (Table 2) and age-standardized prevalences (Table S1; Figure 1). Annual cascade (Table 3) and sex/area gaps (Table 4); inequality (ECI) in Figure S3. 2030 projections and gaps vs 80–80–80. |
| **17** | Other analyses: Report other analyses done (e.g., analyses of subgroups and interactions, and sensitivity analyses). | Results: cascade stratified by sex (Table S2; Figure 2), age (Table S3), wealth quintile (Table S4), area (Table S5), and insurance type (Figure S4). Joinpoint by series (Table S5). Trends in prehypertensive categories (Table S6). Department-level heterogeneity via heatmap (Figure S5). |
| **DISCUSSION** | | |
| **18** | Key results: Summarize key results with reference to study objectives. | Discussion, “Key findings”: the cascade showed low/stagnant performance in 2014–2024, with diagnosis as the main bottleneck; comparison under dual criteria showed stable Hypertension ESH prevalence, while Hypertension ACC/AHA and discordance (reclassified 130–139/80–89 without diagnosis) increased; no joinpoints were identified. Projections to 2030 show gaps of ~26–31 pp versus 80–80–80 targets. |
| **19** | Limitations: Discuss limitations of the study, taking into account sources of potential bias or imprecision. Discuss both direction and magnitude of any potential bias. | Discussion, “Limitations”: (1) repeated cross-sectional design (no causality or individual trajectories); (2) BP measured at a single visit (mean of two readings) without clinical confirmation on multiple occasions; (3) awareness and treatment based on self-report (potential differential error); (4) ENDES does not capture adherence, drug type/dose, or number of medications; (5) reduced 2020 sample size and potential bias from complete-case analytic subpopulation; (6) limited power/multiplicity concerns in joinpoint with 11 annual points; (7) 2030 projections assume continuity of trends. |
| **20** | Interpretation: Give a cautious overall interpretation of results considering objectives, limitations, multiplicity of analyses, results from similar studies, and other relevant evidence. | Discussion, “Comparison with other studies” and “Public health implications”: cautious interpretation in relation to prior national studies (Villarreal-Zegarra; Carrillo-Larco; Diaz-Arocutipa) and international evidence (NCD-RisC, multicountry study in 44 low-/middle-income countries, HEARTS implementation, NHANES 2021–2023, South Korea). The discussion addresses the effect of different diagnostic thresholds (ESH 2023 vs ACC/AHA 2025/2017), early losses (diagnosis and treatment initiation/continuity), socioeconomic inequalities, and implications for strengthening primary care and monitoring toward 2030. |
| **21** | Generalisability: Discuss the generalisability (external validity) of the study results. | Methods, “Data source”: ENDES has national representativeness, by area, natural region, and department. An explicit section on generalisability/external validity is not identified in the Discussion; to align with STROBE, a sentence could be added in the Discussion or Conclusions indicating that results are generalisable to adults (≥18 years) living in private households in Peru (per the ENDES design). |
| **OTHER INFORMATION** | | |
| **22** | Funding: Give the source of funding and the role of the funders for the present study. | The study was funded by the Vice-Rectory for Research of the Universidad Nacional Toribio Rodríguez de Mendoza de Amazonas. The funder had no role in study design, data analysis, interpretation, or manuscript preparation. |
